# CAR-M2 immunotherapy resolves renal fibrosis via revascularization and apoptosis of profibrotic Cxcr2^+^ endothelial cells

**DOI:** 10.1016/j.xcrm.2026.102698

**Published:** 2026-03-25

**Authors:** Wenyan Zhao, Xin Zhou, Xingli Zhao, Hao Tian, Yang Su, Shanlan Zhao, Min Liu, Qiao Zhang, Lin Chen, Xiaochen Li, Di Liu, Junxuan Li, Lang Li, Yanhong Wang, Xingtong Li, Jin Yan, Wen Chen, Bing Liu, Chuhong Zhu, Wen Zeng

**Affiliations:** 1 Department of Cell Biology, Army Medical University, Chongqing 400038, China; 2 Department of Pain and Rehabilitation, Xinqiao Hospital, Third Military Medical University, Chongqing 400038, China; 3 Jinfeng Laboratory, Chongqing 401329, China; 4 Department of Pathology, The 8th Medical Center, Chinese PLA General Hospital, Beijing 100091, China; 5 State Key Laboratory of Experimental Hematology, Fifth Medical Center of Chinese PLA General Hospital, Beijing, China; 6 Department of Anatomy, Engineering Research Center for Organ Intelligent Biological Manufacturing of Chongqing, Key Lab for Biomechanics and Tissue Engineering of Chongqing, Third Military Medical University, Chongqing 400038, China; 7 Engineering Research Center of Tissue and Organ Regeneration and Manufacturing, Ministry of Education, Chongqing 400038, China; 8 State Key Laboratory of Trauma and Chemical Poisoning, Chongqing, China

**Keywords:** CAR-M2, HAMA-CS hydrogel, revascularization, Cxcr2^+^ ECs, FAP^+^ fibroblasts, renal fibrosis

## Abstract

Renal fibrosis is a common outcome of chronic kidney disease (CKD), forming a fibrotic niche characterized by fibroblast activation and vascular rarefaction. Currently, there are no effective treatment strategies targeting fibrotic niche. Here, we show that chimeric antigen receptor-modified M2 macrophages (CAR-M2) targeting FAP and secreting interleukin (IL)-4 are delivered via an injectable HAMA-CS hydrogel beneath the renal subcapsule and attenuate renal fibrosis while promoting renal revascularization. The single-cell RNA sequencing reveals the heterogeneity and interaction of stroma and endothelial cells (ECs). A fibrosis-related Cxcr2^+^ EC subset is identified, and its specific depletion effectively mitigates renal fibrosis. Further results reveal that CAR-M2 can release matrix metalloproteinase 2 (MMP2) in close proximity to activate retinoid X receptor alpha (Rxra) in the Cxcr2^+^ ECs and further triggers its mitochondrial autophagy, leading to apoptosis. Our research provides innovative strategies and proof of principle for the immunotherapy of organ fibrosis.

## Highlights

• HAMA-CS hydrogel encapsulating CAR-M2 alleviates renal fibrosis via subcapsular delivery
• CAR-M2 targets FAP^+^ fibroblasts for phagocytosis and promotes kidney revascularization
• Pro-fibrotic Cxcr2^+^ ECs were identified that regulated renal fibrosis via Lcn2
• CAR-M2-secreted MMP2 activates RXRA, leading to apoptosis of profibrotic Cxcr2^+^ ECs

## Graphical abstract

**Figure:**
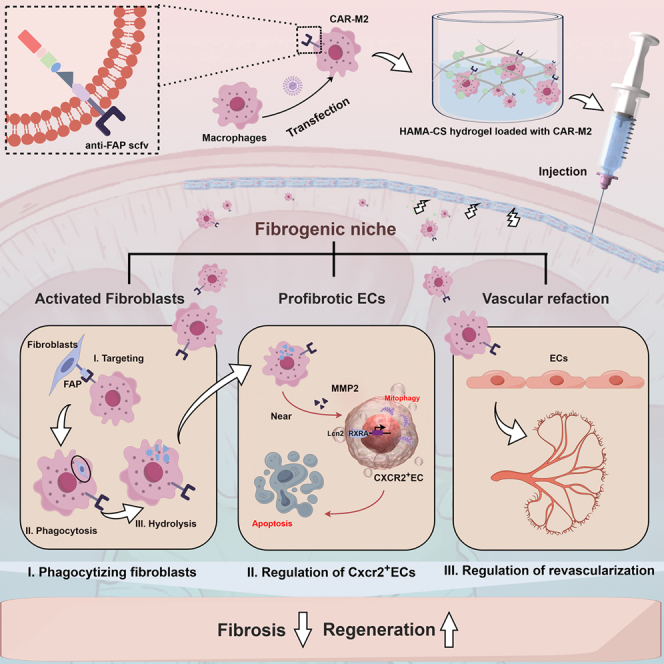

## Teaser

Zhao et al. engineer CAR-M2 macrophages delivered via HAMA-CS hydrogel to target renal fibrosis. The therapy clears FAP^+^ fibroblasts, promotes revascularization, and induces apoptosis of profibrotic Cxcr2^+^ endothelial cells via MMP2-RXRA signaling, achieving potent anti-fibrotic efficacy in kidney models.

## Introduction

Chronic kidney disease (CKD) ranks as the fourth leading cause of mortality, impacting over 10% of the global population.^1^^,^^2^ Fibrosis represents the consequence of renal damage, and its severity is inextricably linked to clinical outcomes.^3^ Until now, there are still no approved available therapies for renal fibrosis. Renal fibrosis is accompanied by the aberrant activation of fibroblasts and rarefaction of blood vessels.^4^^,^^5^^,^^6^ Kidney fibrosis involves the dynamic interactions of various cells, including immune cells, stromal cells, and endothelial cells (ECs), among others. Macrophages regulate regeneration and fibrosis through the interaction with other cell types, encompassing the clearance of apoptotic myofibroblasts and the regulation of the endothelial microenvironment.^7^ Moreover, M2 macrophages promote tissue angiogenesis and participate in the reparative processes of tissue remodeling.^8^^,^^9^ Consequently, the significance of macrophages within the fibrotic niche may play a crucial role in regulating both the fibrosis and the vascular microenvironment.

Chimeric antigen receptor (CAR) macrophages (CAR-Ms) are utilized in the treatment of solid tumors^10^^,^^11^ due to their superior ability to penetrate and phagocytose tissue cells compared with CAR-T.^11^^,^^12^^,^^13^ CAR-immune cells have been used in non-neoplastic disease.^14^^,^^15^ FAP-targeting CAR-M offers a strategy for the treatment of myocardial fibrosis.^16^^,^^17^ Inspired by this, the use of CAR-Ms to target fibrotic cells could represent an effective strategy for treating significant organ fibrosis. To date, in the absence of minimally invasive treatments for renal fibrosis, injected cells are both difficult to target within the parenchymal organ and to enrich in the hostile microenvironment generated by disease foci.^18^ Renal subcapsule drug delivery has been shown to be a safe and effective mode of kidney disease administration.^19^ Hydrogel materials can be injected or implanted in a minimally invasive manner to deliver the therapeutic drug to the target site.^20^^,^^21^^,^^22^ Plastic, low-swelling hydrogels that encapsulate cells and are delivered via renal subcapsular injection could represent a minimally invasive and effective strategy for renal fibrosis cell therapy. Among them, hyaluronic acid methacrylate (HAMA) hydrogel has been extensively studied for cell loading and wound healing.^23^ The combination of chitosan (CS) with HAMA is better suited to meet the low-swelling requirements beneath the renal subcapsule while preserving its plasticity.^24^

The renal fibrosis niche is an unfavorable microenvironment for ECs, causing microvascular rarefaction in the kidney.^25^ During the process of fibrosis, EC subsets exhibiting pro-fibrotic molecular phenotypes will emerge. This specific subset of ECs, in turn, recruits profibrotic immune cells or triggers the activation of fibroblasts, thereby accelerating the progression of fibrosis.^26^ It has been demonstrated that renal capillary ECs can participate in renal fibrosis by undergoing a reduction in number or by transforming into mesenchymal cells.^27^ Exploring and regulating this specific subset of pro-fibrotic ECs could be an effective strategy to alleviate fibrosis. However, the specific EC subsets acting on kidney fibrosis and its mechanism are currently unknown.

In this study, our objective was to alleviate renal fibrosis using a biocompatible HAMA-CS hydrogel loading with CAR-M2. CAR-M2 can effectively target FAP^+^ fibroblasts, which are enriched in fibrotic kidney, thereby promoting renal vascular reconstruction. The heterogeneity and interaction of stroma and EC were discovered by performing single-cell RNA sequencing (scRNA-seq). Fibrosis-related Cxcr2^+^ ECs were identified. Combined with analysis of CKD samples, we further explored the mechanism of CAR-M2 indirectly alleviating fibrosis through the regulation of Cxcr2^+^ ECs. Our research provides an effective cell-targeted therapy strategy for addressing renal fibrosis.

## Results

### Construction of CAR-M2 with FAP-specific fibroblasts targeting and phagocytosis

**Figure 1:**
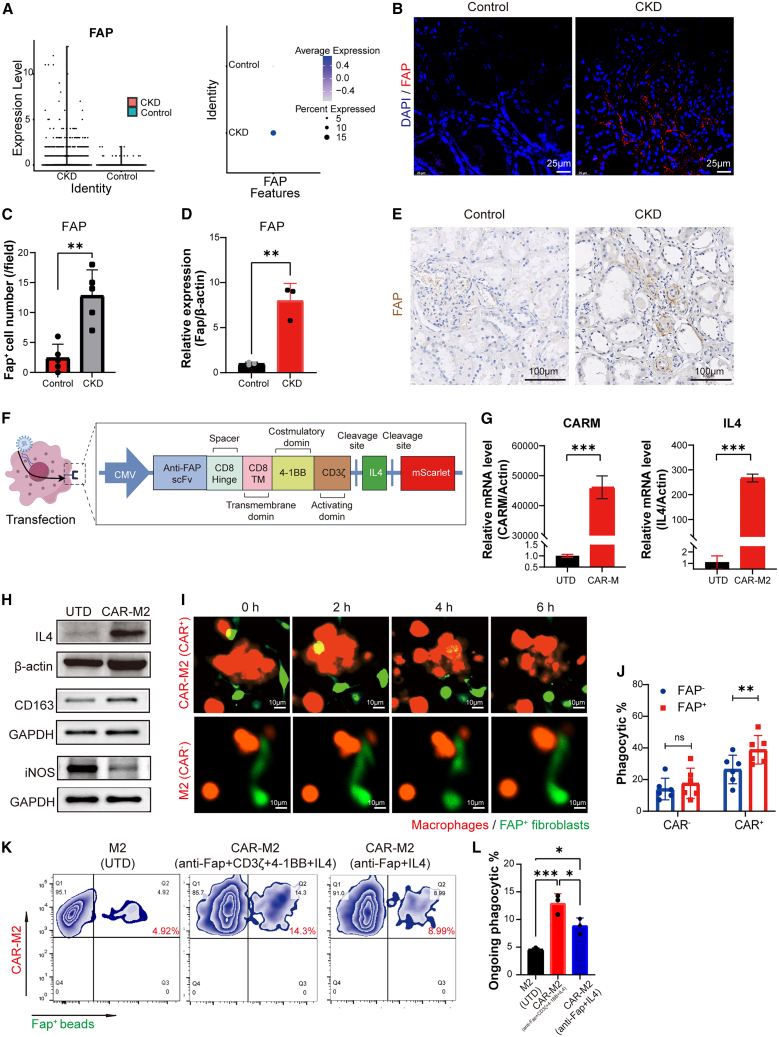
CAR-M2 are engineered to specifically target and engulf renal fibrosis-associated FAP^+^ fibroblasts (A–E) *FAP* expression in CKD and healthy kidney tissues analyzed by scRNA-seq (A), immunofluorescence staining (B and C), qPCR (D), and immunohistochemical staining (E). Scale bars: 25 μm in (B) and 100 μm in (E). (F) Transgene construct lentiviral sequences to express anti-FAP CAR and IL-4. (G) Upregulated CAR and IL-4 mRNA expression after IL-4-CAR transduction to M2 macrophages *in vitro*, evaluated using qPCR (*n* = 3, two-tailed Student’s *t* test). (H) Western blot measured the protein expression levels of IL-4, M2 marker CD163, and M1 marker iNOS after CAR-M2 constructs at 72 h. (I) Representative images of CAR-M2 and M2 co-cultured with FAP^+^ human fibroblasts at 24 h, observed by live-cell microscopy. Scale bars, 10 μm. (J) Percentage of phagocytosis by CAR-M2, represented by the reduction in FAP^+^ human renal fibroblasts after 24 h, as observed by live-cell microscopy (*n* = 6, two-sided *t* test). (K) Flow cytometry characterizes the difference in phagocytic efficiency of CAR-M2 with the intracellular activation domains (4-1BB and CD3ζ) versus CAR-M2 without the intracellular activation domains (CAR-M2 co-culture with FAP^+^ beads). (L) Statistical analysis of the results in (K), *n* = 3. ∗*p* < 0.05, ∗∗*p* < 0.01 and ∗∗∗*p* < 0.001. See also Figures S1 and S2.

Endogenous proteins expressed by disease-associated cells can be used as a specific targeted antigen.^14^^,^^15^ The gene expression patterns revealed by scRNA-seq datasets from mice with renal fibrosis and human CKD^3^^,^^28^ demonstrated the upregulation of FAP in human CKD samples (Figure 1A) and in mice with unilateral ureteral obstruction (UUO) (Figure S1A). FAP was robustly detected in the fibrotic kidney tissue from both human CKD samples (Figures 1B–1E; S2A) and UUO mice (Figures S1B and S2B). Thus, FAP was regarded as an ideal antigen for targeting activated fibroblasts in fibrotic kidney.

The CAR-M2 lentivirus was constructed by cloning the anti-FAP CAR-IL-4-M2 gene into the GV400 vector. The anti-FAP CAR-IL-4-M2 consists of an anti-FAP single-chain variable fragment, a CD8 hinge domain, a CD8 transmembrane domain, a CD137 cytoplasmic domain, a CD3ζ cytoplasmic domain, an interleukin (IL)-4-secreting domain, and a red fluorescent protein (Figure 1F). The CD3ζ intracellular domain of the CAR-M stimulates phagocytosis-related signaling in macrophages.^29^ The IL-4 gene was utilized for the long-term maintenance of the immunosuppressive M2 phenotype.^12^^,^^30^ The expression of both CAR and IL-4 mRNA was significantly elevated in M2 macrophages following transduction with IL-4-CAR (Figure 1G). The transduction efficiency is indicated by the percentage of red fluorescent protein (mScarlet) in transduced cells (Figure S1C). The viability of the cells remained unaffected following transduction and expression of the CAR-M2 lentivirus (Figure S1D). Cells that were successfully transduced with CAR-M2 were isolated for further investigation (Figure S1E). The M2 bone marrow-derived macrophages were transduced with a CAR-M2 lentivirus, which significantly upregulated the expression of anti-inflammatory M2 macrophage markers (CD163, arginase-1, and IL-10) and downregulated the expression of inflammatory M1 markers (CD86, iNOS, and interferon-γ) (Figures 1H and S1F–S1I).^31^^,^^32^

Renal fibrosis is often accompanied by the abnormal activation of fibroblasts.^33^ Transforming growth factor β1 (TGF-β1) induces the differentiation of fibroblasts to myofibroblasts *in vitro.*^34^ Thus, an *in vitro* model of renal fibrosis was established by transducing primary human renal fibroblasts with FAP lentivirus. The transduced cells exhibited FAP expression levels comparable to those of fibroblasts induced by TGF-β1 (Figures S2C–S2F), thereby demonstrating the viability of an *in vitro* fibrotic cell model based on FAP lentiviral-transduced fibroblasts. Co-cultures of CAR^+^- or CAR^−^-treated THP-1-induced M2 macrophages with FAP^+^ or FAP^−^ human fibroblasts were established at a 1:1 ratio for 24 h. Figure 1I displays the representative images of CAR-M2 or M2 co-cultured with FAP^+^ human fibroblasts for 6 h. A significant decrease in GFP-labeled FAP^+^ human fibroblasts was observed (Figure 1J; Videos S1 and S2). The CAR-M2 (CAR^+^ group) exhibited a 2-fold increase in antigen-specific phagocytic activity, as compared to those macrophages transduced solely with mScarlet (CAR^−^ group). Furthermore, CAR-M2-transduced M2 macrophages exhibited a higher rate of phagocytosis of FAP^+^ human fibroblasts compared to those transduced with FAP^−^ cells, achieving a nearly 40% phagocytic rate. No such difference was observed in the CAR^−^ group, indicating that CAR-M2 not only exhibits specific phagocytosis of FAP^+^ targets but also displays enhanced basal phagocytic activity even in the absence of the target antigen (Figure 1J). Additionally, flow cytometry analysis using FAP^+^ beads in place of FAP^+^ fibroblasts showed that the phagocytic efficiency of CAR-M2 macrophages containing the costimulatory domain was enhanced compared to both those without the costimulatory domain and non-transduced M2 macrophages (Figures 1K and 1L). The induction and construction of CAR-M2 by human pluripotent stem cells (hPSC) have also been successfully accomplished (Figures S2G–S2K).

Video S1. CAR-M2 target and phagocytose FAP^+^ fibroblasts

Video S2. CAR-M2 target and phagocytose FAP^+^ fibroblasts

### Engineered HAMA-CS hydrogel renal subcapsule delivery of CAR-M2 alleviates mice fibrosis

**Figure 2:**
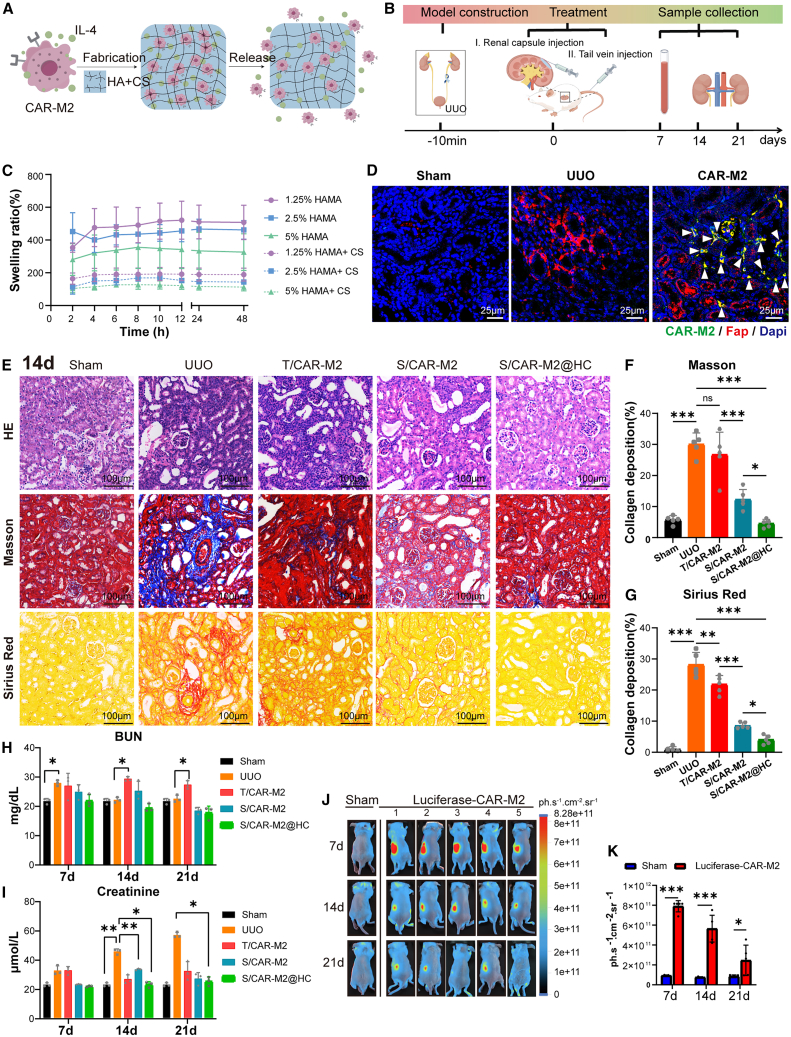
HAMA-CS hydrogel *in situ* sustained-release CAR-M2 attenuates renal fibrosis in UUO mice (A) Scheme of the construction and implantation of the engineered HAMA-CS hydrogel. (B) Flow diagram of the treatment regimen against renal fibrosis in UUO mice. (C) Swelling ratio curve of HAMA and HAMA-CS at 1.25, 2.5, and 5% w/v concentration (*n* = 3). (D) Fluorescence images of co-localized FAP^+^ fibroblast (red) and CAR-M2 (green) in kidney. Nuclei were stained with DAPI (blue). Scale bars, 25 μm. Arrowheads indicate co-localization of CAR-M2 with FAP^+^ cells. (E–G) Representative H&E, Masson, and Sirius red staining images of mice renal tissue sections following the injection of PBS into the renal subcapsule (Sham and UUO), CAR-M2 solution injection into the tail vein (T/CAR-M2), CAR-M2 solution local renal subcapsule injection (S/CAR-M2), and CAR-M2-loaded hydrogel injected into the renal subcapsule (S/CAR-M2@HC). (E) Detection at day 14 after UUO. Scale bars, 100 μm. Average collagen deposition density by Masson (F) and Sirius red (G) staining (*n* = 5, one-way ANOVA). (H and I) Changes of blood urea nitrogen (H) and creatinine (I) levels at 14 days after different CAR-M2 treatment (*n* = 3, two-way ANOVA). (J) Luciferase labeling of CAR-M2 was conducted and the lifespan of these subcapsular-injected CAR-M2 was detected by *in vivo* fluorescence imaging (*n* = 5). (K) Total radiant efficiency normalized of (J) by manual measurements over the fluorescence region of interest and by the initial signal intensity (*n* = 5, two-way ANOVA). ∗*p* < 0.05, ∗∗*p* < 0.01, and ∗∗∗*p* < 0.001. See also Figures S3 and S4.

The hydrogel was generated using the HAMA that can be cross-linked by ultraviolet irradiation.^23^^,^^35^^,^^36^ CAR-M2 targeting the FAP antigen and releasing IL-4 was loaded into the 2.5% HAMA-1% CS hydrogel (2.5% HAMA-CS) (Figure 2A; Video S3). Local intervention was started immediately after UUO surgery, and the serum and renal tissue samples were collected on days 7, 14, and 21 for kidney functional, histological, and immunohistochemical analyses (Figure 2B). The Sham group was only subjected to ureteral isolation. The UUO group was treated only with renal subcapsule injection of PBS after UUO surgery. The CAR-M2 group was treated with HAMA-CS hydrogel loading CAR-M2 by renal subcapsular injection. The freeze-dried HAMA hydrogel had a tightly arranged porous structure with sizes ranging from 20 to 100 μm (Figures S3A and S3B). The high swelling property may increase the hardness of the extracellular matrix (ECM) in the visceral parenchyma and accelerate the process of renal fibrosis.^37^^,^^38^ The swelling property of HAMA needed to be improved while retaining its plasticity for an effective release of the cells into the kidney using a hydrogel with a good plasticity (Figure S3C). Therefore, as a commonly used natural biomaterial with good swelling performance, CS was incorporated into the HAMA (HAMA-CS) to reduce the swelling ability of HAMA.^24^ The swelling rate of HAMA-CS was about 50% of that of HAMA with the same concentration (Figure 2C).

Video S3. Construction and application of CAR-M2-loaded hydrogels

The degradation of the HAMA-CS hydrogel was assessed *in vitro* (Figure S3D), resulting in a 2-fold degradation rate compared with HAMA hydrogel in the same time and concentration. The HAMA-CS hydrogel released CAR-M2 in a sustained manner for more than 21 days (Figure S3E). The location or distribution of CAR-M2 was visualized by the red fluorescence (expression of CAR-M2) and scanning electron microscope (Figures S3F and S3G). As regards its biocompatibility, the hydrogel formulation did not cause any observable toxic effects on survival of CAR-M2 (Figure S3H). The degradation of HAMA-CS hydrogel was assessed *in vivo* (Figures S3I–S3K), demonstrating a gradual degradation over 21 days and meeting the sustained release criteria for CAR-M2. Taken together, these results demonstrated that the HAMA-CS hydrogel acted as an ideal vehicle for CAR-M2 delivery. The persistence, biodistribution, and biosafety of CAR-M2 cells were investigated in the UUO mouse model after different administration manners. The CAR-M2 group was subdivided based on different modes of administration; the T/CAR-M2 group was treated with CAR-M2 by tail vein injection. CAR-M2 was injected in the renal subcapsular area, named the S/CAR-M2 group, and the S/CAR-M2@HC group was treated with HAMA-CS hydrogel loading CAR-M2 by renal subcapsular injection. The DiR-labeled CAR-M2 persisted for at least 21 days *in vivo* in the S/CAR-M2@HC group. The kidney in all groups was the primary tissue where CAR-M2 was accumulated after administration, suggesting the kidney-targeted biodistribution of CAR-M2 (Figure S3L). A more than 4 × 10^10^ ph.s^−1^.cm^−1^.sr^−1^ radiance in the kidney was observed in the S/CAR-M2@HC group (Figure S3M), indicating that HAMA-CS hydrogel remained in the kidney and performed sustained release of CAR-M2 *in situ*. Furthermore, the co-localization of CAR-M2 and FAP^+^ fibroblasts was found in the CAR-M2 treatment group (Figure 2D), and CAR-M2 was able to migrate into the kidney tissues after UUO (Figure S3N). As regards its biosafety, the HAMA-CS hydrogel loaded with CAR-M2 did not cause any toxic effects on injected mice (Figures S3O–S3R).

A mouse model with renal fibrosis was established by performing UUO to explore the therapeutic efficacy of CAR-M2 loaded in HAMA-CS hydrogel. The administration method and naming of the treatment groups are shown in the Figure S3L. To show the predominance of CAR-M2, the control groups via HAMA-CS renal subcapsule delivery of M2 (S/M2@HC) and codelivery of IL-4 with CARM (without IL-4) (S/IL-4+CARM@HC) were established. After surgery, the UUO group showed some renal tubular epithelial cell vacuoles, renal tubule dilatation, clearly widened tubular space, and inflammatory cell infiltration in the interstitial space and significantly increased collagen deposition, compared with the Sham group (Figures 2E–2G and S4A–S4D).

The groups treated with CAR-M2 showed a positive therapeutic effect with reduced morphological changes in the kidney, whereas the S/CAR-M2@HC group showed a decreased inflammatory cell infiltration and renal tubular epithelial cell vacuoles in the kidney compared to the UUO group (Figures 2E, S4A, and S4B). The collagen deposition in CAR-M2 and M2 treatment groups showed remission at various levels. The S/CAR-M2@HC group showed the best therapeutic effect—the collagen deposition was only 30% of that in the UUO group at the same time (Figures 2F, 2G, S4C, and S4D). The CAR-M2 injection via tail vein induced weak inhibitory effect on fibrosis. Both type I and type III collagen were significantly reduced in UUO mice after CAR-M2 treatment (Figures S4E–S4G). Serum creatinine and blood urine nitrogen, the indexes of renal function were evaluated, revealing nearly 30% functional improvement in the CAR-M2 treatment group compared with the UUO group, indicating that the overall renal metabolism remained normal and the UUO model has little effect on renal function (Figures 2H and 2I). A luciferase-labeled CAR-M2 could survive for a long time and be persistently released from HAMA-CS hydrogel with 21 days in the kidney after injury (Figures 2J and 2K).

### Cellular profiling and heterogeneity of stroma cells following CAR-M2 treatment

**Figure 3:**
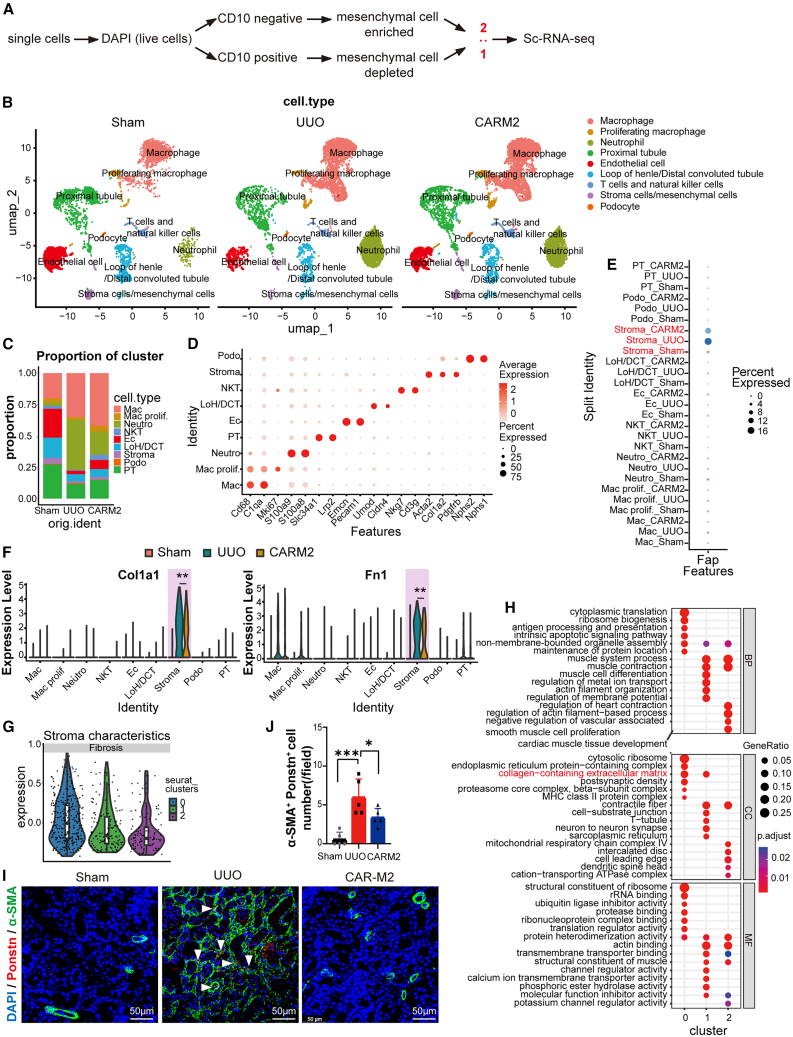
The scRNA-seq offered a comprehensive cellular profiling and illustrated a remission in fibrosis subsequent to CAR-M2 therapy (A) Cell sorting protocol performed before single-cell sequencing. (B) UMAP showing cell clusters of the Sham, UUO, and CAR-M2 mice kidney. Cells colored by cell type. (C) Bar plots displaying the proportional abundance of each cell type in each group. Mac, macrophage; Mac prolif., proliferating macrophage with high ki67 expression; Neutro, neutrophil; PT, proximal tubule; LoH, loop of Henle; DCT, distal convoluted tubule; NKT, T cells and natural killer cells; Stroma, stroma cells/mesenchymal cells; Podo, podocyte. Different colors represent different cell types. (D) Dot plots showing the expression of cell cluster marker genes. (E) Dot plots showing the *Fap* expression in different groups and cell types. (F) Quantification of fibrosis-related gene expression in different groups and cell types. (one-way ANOVA). (G) Functional scoring of fibrosis-related gene sets in 3 stromal cell clusters. (H) Gene Ontology (GO) enrichment analysis of differentially expressed genes among 3 stromal cell clusters. (I and J) Immunofluorescence co-staining and quantitative analysis of the cluster 0 markers Postn with α-SMA. Scale bars, 50 μm (*n* = 5, one-way ANOVA). Arrowheads indicate the double positive cells. ∗*p* < 0.05 and ∗∗∗*p* < 0.001. See also Figure S4.

A single-cell map of the kidney was generated. Mice were subjected to different treatments, including Sham, UUO, and treatment by HAMA-CS hydrogel loading CAR-M2 (the S/CAR-M2@HC group in the aforementioned animal treatment, hereinafter referred to as the CAR-M2 group). Since more than 80% of renal cortical cells are proximal tubular epithelial cells, non-proximal tubular cells (CD10^−^) and CD10^+^ proximal tubular cells were separated by flow cytometry to accurately detect smaller cell populations and map of the kidney.^39^ CD10^+^ and CD10^−^ cells were mixed in a 1:2 ratio before scRNA-seq (Figure 3A). Nine major cell types and their proportions were identified after 10× single-cell sequencing (Figures 3B and 3C). Bubble plots showed the marker genes for each cell population (Figure 3D). The expression of *Fap* was upregulated in the renal stroma cells of the UUO group, but it was decreased in the CAR-M2 group (Figure 3E).

Subsequently, the expressions of common fibrogenic niche markers were evaluated (Figures 3F and S4H). Among them, *Loxl2*, *Lbp*, *Fn1*, *Col1a1*, and *Fbn1* were highly expressed in the renal stroma cells, participating in ECM deposition, releasing fibrotic factors, and accelerating the injury and apoptosis of renal parenchymal cells.^25^^,^^40^ The expression of *S100a8* increased in various cell components of the kidney and played important roles in tubular cell injury and promoting inflammatory cell infiltration.^40^^,^^41^ The expression of these fibrogenic markers was significantly reduced in CAR-M2 group. *Gpx3*, as a gene highly expressed in normal renal tubular epithelial cells, was an important indicator of renal oxidative stress level and fibrosis progression. The recovery of *Gpx3* expression level after CAR-M2 therapy also suggested a good prognosis for the kidneys.^42^

Three stroma cell subclusters (clusters 0, 1, and 2) were used to further elucidate the regulatory effect of CAR-M2 on fibrosis (Figure S4I). Cluster 0 showed a dynamic change in proportion, with an initial increase and subsequent decrease in CAR-M2 group (Figure S4J). Cluster 0 also showed an increased expression of inflammatory and fibrosis-related genes (*Col1a1, Acta2, Pdgfra, Postn,* and *Dcn*) (Figures S4K and S4L).^25^^,^^43^ The results of fibrosis scores on the three stroma cells further confirmed the highest fibrosis score of cluster 0 stroma cells (Figure 3G). Cluster 0 stroma cells played a crucial role in promoting cell apoptosis, accelerating the deposition of ECM, and disrupting the homeostasis of the renal microenvironment (Figure 3H). Finally, the result of immunofluorescence staining (Figures 3I and 3J) confirmed that *postn* (the marker gene of fibrosis-related fibroblasts) is highly expressed in cluster 0 stroma cells.

### CAR-M2 promotes renal revascularization by affecting the interaction between macrophages and ECs

**Figure 4:**
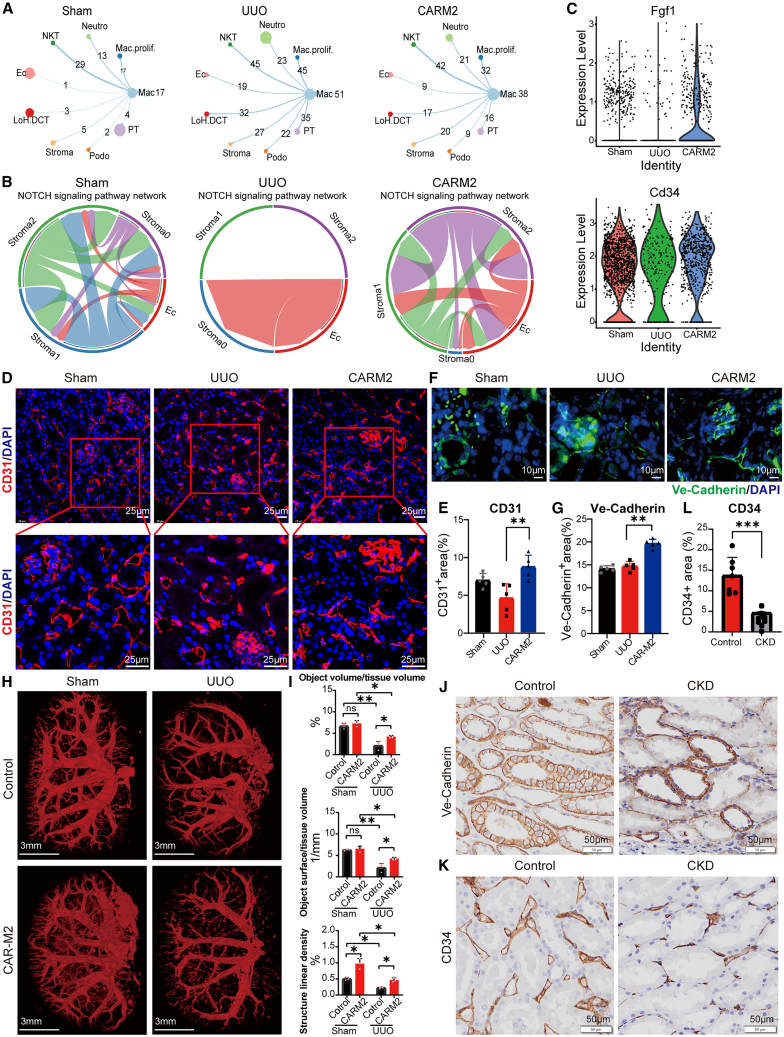
CAR-M2 enhanced renal revascularization following renal fibrosis (A) Cell-cell interactions between macrophages and other cell types in each group. (B) Changes in cell-cell interactions in the Notch signaling pathways related to angiogenesis in three groups. (C) Expressions of angiogenesis marker genes (*Fgf1* and *Cd34*) in three groups. (D) Immunofluorescence staining of CD31 (red) and DAPI (blue) on a tissue section collected from Sham, UUO, and CAR-M2 at 14 days. Scale bars, 25 μm. (E) The morphometric quantification of CD31 area per high-power field (HPF) (*n* = 5). (F) Immunofluorescence staining of Ve-Cadherin (green) and DAPI (blue) on a tissue section collected from three groups. Scale bars, 10 μm. (G) Cartogram showing the morphometric quantification of Ve-Cadherin area per HPF (*n* = 5). (H) Representative *ex vivo* micro-computed tomography (mCT) images showing 3-dimensional (3D) visualization after Microfil perfusion on day 14 after Sham, UUO, and CAR-M2 treatment. Scale bars, 3 mm. (I) mCT-based quantification of vascular volume, vascular surface area, and vascular density. *n* = 3. (J–L) Expressions and structure of Ve-Cadherin and CD34 in tissue sections from human normal and CKD groups detected by immunohistochemistry staining (*n* = 7). Scale bars, 50 μm. ∗*p* < 0.05, ∗∗*p* < 0.01, ∗∗∗*p* < 0.001, ns, not significant (one-way ANOVA). See also Figure S5.

The proportion of ECs was significantly increased in CAR-M2 treatment mice compared with the UUO mice. Moreover, a change in the interaction between macrophages and ECs was discovered (Figure 4A). Thus, we evaluated whether changes of this interaction in the CAR-M2 group regulated the microvascular network reconstruction after renal fibrosis. The analysis of angiogenesis-related NOTCH and VEGF signaling pathway in the three groups (Sham, UUO surgery, and CAR-M2 group) showed a great diversity (Figures 4B and S5A). Notch signaling is important in angiogenesis for both the embryo and adult.^44^ The promotion effect on EC tube formation and proliferation by CAR-M2 was demonstrated *in vitro* (Figures S5B–S5D), revealing its role in angiogenesis.

The expressions of *Fgf1* and *Cd34* were also upregulated after treatment with CAR-M2 (Figure 4C), which were the commonly representative markers of angiogenesis.^45^^,^^46^ The rarefaction of CD31^+^ peritubular capillaries (Figures 4D and 4E) was more serious in UUO kidneys. Vascular endothelial cadherin (Ve-Cadherin) is a component of EC-to-cell adherent junctions, and it is required for the maintenance of vascular integrity.^47^ Consistent with CD31, the CAR-M2 group showed a stronger junctional staining for Ve-Cadherin (Figures 4F and 4G). The Sham and CAR-M2 groups showed distinct Ve-Cadherin continuous staining at cell borders. Micro-computed tomography after Microfil injection revealed a significant preservation or reconstruction of renal vasculature in kidneys of CAR-M2 treatment mice (Figures 4H and 4I). The results above indicated that CAR-M2 alleviated renal fibrosis and concurrently regulated ECs to promote renal revascularization.

The abundance of Ve-Cadherin and CD34 protein was investigated in healthy and CKD human kidney tissues. Consistent with the results in mice, the healthy samples (control group) showed a stronger junctional cell staining and a clearer structure of Ve-Cadherin compared to those in the samples of CKD (Figure 4J). A higher expression of CD34 in healthy tissues compared with that in CKD tissues confirmed the less vascular network and angiogenesis in the CKD tissues (Figures 4K and 4L).

### CAR-M2 indirectly modulates renal fibrosis by influencing Cxcr2^+^ ECs

**Figure 5:**
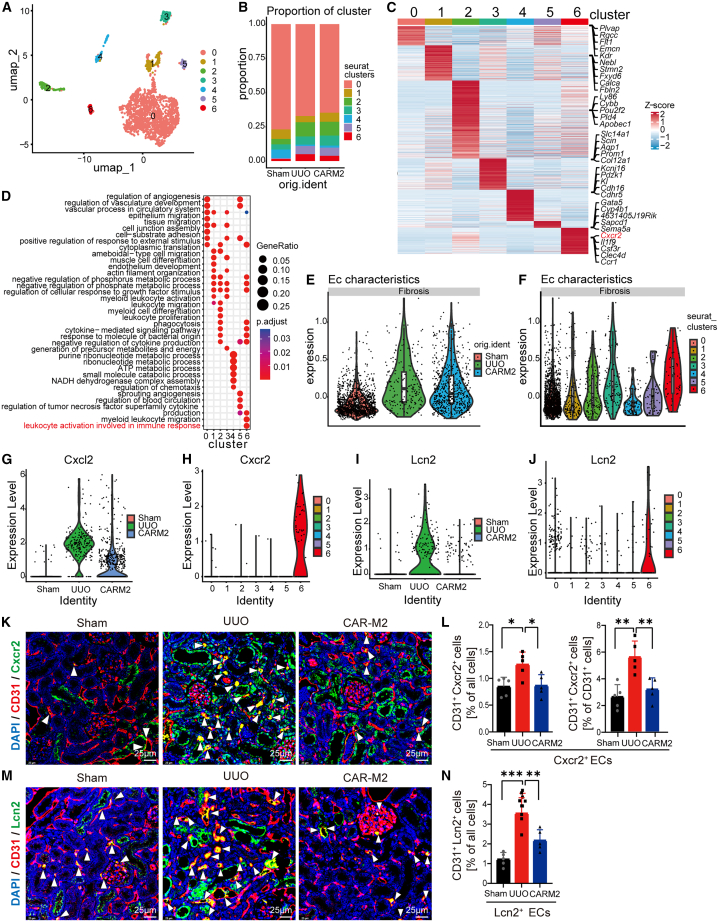
CAR-M2 regulated mice renal fibrosis through profibrotic Cxcr2^+^ ECs (A) UMAP showing the subclusters of ECs in the kidney. (B) Bar plots displaying the proportional abundance of each EC subcluster in each condition. (C) Heatmap showing representative gene expression of each EC subcluster. (D) GO enrichment analysis on upregulated differentially expressed genes of each EC subcluster. (E) Fibrosis-related gene score in Sham, UUO, and CAR-M2 groups. (F) Fibrosis-related gene score of 7 EC clusters. (G–J) *Cxcl2* was highly expressed in UUO group (G), and its receptor gene Cxcr2 was highly expressed in the cluster 6 of ECs (H). Similarly, *Lcn2* was specific highly expressed in UUO group (I) and cluster 6 ECs (J). (K–N) Immunofluorescence co-staining and quantitative analysis of the cluster 6 marker Cxcr2/Lcn2 in CD31. Scale bars, 25 μm. Arrowheads indicate the double positive cells. ∗*p* < 0.05, ∗∗*p* < 0.01, and ∗∗∗*p* < 0.001 (one-way ANOVA). See also Figure S5.

As mentioned above, ECs showed strong regulation on the stroma cluster 0. Therefore, the crosstalk between ECs and renal fibrosis was assessed. Single-cell analysis of ECs revealed 7 cell clusters (Figure 5A). The interaction between fibrosis-related stroma cluster 0 and EC cluster 6 decreased after UUO and returned to the value of the Sham group after CAR-M2 treatment. No TGF-β signaling regulation was observed between EC cluster 6 and stroma cluster 0 in the UUO group, while the other two groups showed a rich and similar regulatory network (Figures S5E–S5G), suggesting that EC cluster 6 influenced the progression of renal fibrosis through the interaction with stroma cells. EC cluster 6 increased in the UUO group and decreased after CAR-M2 treatment (Figure 5B). EC cluster 6 was characterized by a strong expression of *Cxcr2*, *Il1f9*, *Csf3r*, *Clec4d*, and *Ccr1* (Figure 5C), and it was mainly involved in the activation of leukocytes related to immune response, promoting the migration of myeloid leukocytes (Figure 5D). Previous studies found that the process of renal fibrosis was accelerated by the recruitment and activation of leukocytes, and the inhibition of this process prevents fibrosis.^48^^,^^49^ In particular, Cxcr2 can drive renal fibrosis by inducing mitochondrial dysfunction in tubular cells.^50^ Based on the top genes and functions of the EC cluster 6, it has been named Cxcr2^+^ ECs. Fibrosis-related scores increased after UUO and decreased after the treatment with CAR-M2 (Figure 5E), demonstrating the potential renal fibrosis-regulating effects of ECs. Among these, the Cxcr2^+^ ECs showed the highest profibrotic gene expression (Figure 5F). The above results suggested the potential regulatory role of Cxcr2^+^ ECs in renal fibrosis. Our further analysis confirmed that the expression of *Cxcl1* was significantly upregulated after UUO (Figure 5G), while its receptor *Cxcr2* was highly expressed in fibrosis-associated Cxcr2^+^ ECs (Figure 5H).

Cxcr2^+^ EC differential genes among the Sham, UUO surgery, and CAR-M2 groups were further analyzed. The genes most significantly upregulated after UUO and downregulated after CAR-M2 treatment were selected by short time-series expression miner analysis. Among these genes, *Lcn2* was highly expressed not only in the fibrotic kidney (Figure 5I) but also in Cxcr2^+^ ECs (Figure 5J). Lcn2 is a biomarker of kidney injury and serves as an early diagnostic factor for CKD.^51^^,^^52^ These results revealed Cxcr2^+^ ECs’ key role in renal fibrosis through Lcn2. The protein expression of Cxcr2 and Lcn2 in ECs was promoted after UUO, and this effect was reversed after CAR-M2 treatment (Figures 5K–5N). Our results implied that Cxcr2^+^ ECs aggravated renal fibrosis through the high expression of fibrosis-related gene *Lcn2*.

### Cxcr2^+^ ECs is implicated in the progression of the renal fibrosis via the profibrotic protein Lcn2

**Figure 6:**
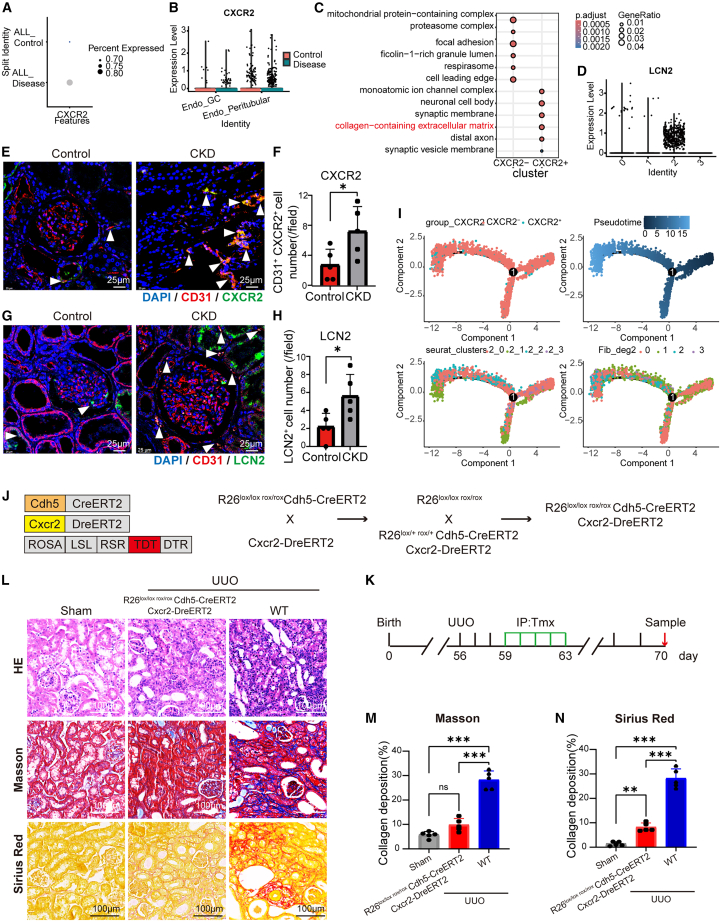
Cxcr2^+^ ECs contribute to renal fibrosis by expressing the profibrotic protein Lcn2 (A) The differential expression of the *CXCR2* gene between healthy and CKD samples in human kidney single-cell sequencing data. (B) The expressions of the *CXCR2* gene in human kidney glomerular endothelial cells (Endo_GC) and peritubular endothelial cells (Endo_Peritubular) under various conditions. (C) GO functional enrichment of differential genes between human kidney CXCR2^+^ endothelial cells (CXCR2^+^ ECs) and CXCR2^−^ endothelial cells (CXCR2^−^ ECs). (D) Expression of *LCN2* among different EC clusters in human kidney single-cell sequencing data. (E–H) In the human kidney, immunofluorescence co-staining is performed to simultaneously visualize CD31 and fibrosis-related genes, such as *CXCR2* (E and F) and *LCN2* (G and H) (*n* = 5). Scale bars, 25 μm. Arrowheads indicate the double positive cells. (I) Pseudotime analysis of the EC with high *CXCR2* expression, showing *CXCR2* expression (top left), pseudotime (top right), subclusters of EC cluster 2 (bottom left), and the degree of fibrosis (bottom right). (J) Specific depletion of Cxcr2^+^ ECs was genetically induced in EC-Cxcr2-DTR mice. (K) Flow diagram of the UUO modeling. (L) H&E, Masson, and Sirius red staining analysis of kidney tissue sections of Sham, UUO, and Cxcr2^+^ EC-specific depletion mice. Detection at day 14. Scale bars, 100 μm. (M and N) Average collagen deposition density (the percentage of positively stained area per HPF) by Masson and Sirius red staining in (L), *n* = 5. ∗*p* < 0.05, ∗∗*p* < 0.01, ∗∗∗*p* < 0.001 and not significant (ns) (one-way ANOVA). See also Figure S6.

Animal experiments demonstrated the important role of Cxcr2^+^ ECs and *Lcn2* gene in renal fibrosis. Human renal fibrosis transcriptome data from public database (GSE211785) were used to analyze whether Cxcr2^+^ ECs are involved in CKD progression.^53^ *Cxcr2* are specifically and highly expressed in the peritubular endothelium of the CKD kidney (Figures 6A and 6B). The cluster enriched Cxcr2^+^ ECs had high *Lcn2* expression and involved in the regulation of fibrosis-associated ECM formation (Figures 6C, 6D, and S6A–S6E). The staining of human kidney samples revealed an increased protein expression of Cxcr2 and Lcn2 in the ECs of CKD (Figures 6E–6H). Cxcr2^+^ ECs emerged and rose as fibrosis state, indicating that Cxcr2^+^ ECs play a key role in the fibrosis process and the human CKD (Figures 6I and S6F–S6I).

The specific depletion of Cxcr2^+^ ECs was achieved by constructing *Cdh5*-CreERT2; *Cxcr2*-DreERT2; Rosa-LSL-RSR-tdTomato-DTR mice (EC-Cxcr2-DTR mice). The renal collagen deposition was significantly reduced to nearly 30% of the original amount in mice depleted of Cxcr2^+^ ECs (Figures 6J–6N). After 14 days of inhibition of Cxcr2 and Lcn2 in UUO mice, renal collagen deposition was significantly downregulated to 50% of the original amount (Figures S6J–S6N). To explore the role of Cxcr2^+^ ECs in the regulation of fibrosis mediated by CAR-M2, we co-cultured ECs overexpressing Cxcr2 with human renal fibroblasts and applied the supernatant of CAR-M2 activated by FAP target protein to ECs to detect fibrosis-related markers (Figure S6O). The results demonstrated that transduction with the lentivirus overexpression plasmid significantly up-regulated Cxcr2 expression in ECs. The addition of CAR-M2 supernatant downregulated the Cxcr2 and Lcn2 expression levels of Cxcr2^+^ ECs (Figure S6P). Further, the expression levels of fibrosis-related genes such as *Col1a1, Col3a1, Pdgfa*, and *Snail* in human renal fibroblasts also showed downregulation upon addition of CAR-M2 supernatant (Figure S6Q). All the above results suggested that CAR-M2 can participate in the progression of renal fibrosis by regulating the Lcn2 expression of Cxcr2^+^ ECs.

### CAR-M2 secretes MMP2 to activate Rxra of Cxcr2^+^ ECs in favor of alleviating fibrosis

**Figure 7:**
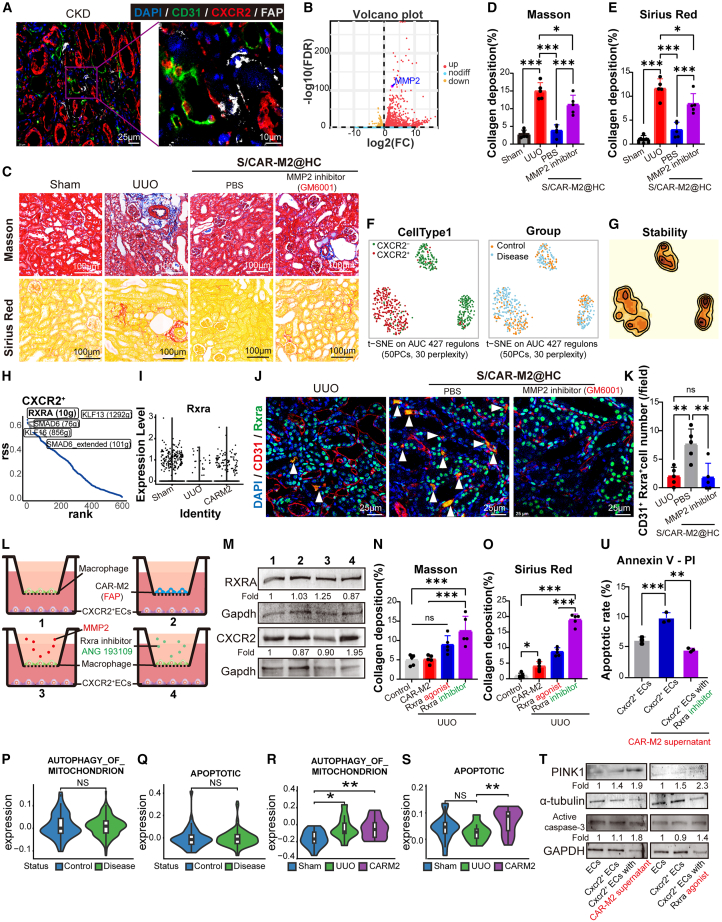
The secretion of MMP2 by CAR-M2 activates the transcription factor Rxra in Cxcr2^+^ ECs (A) Position relationship between CXCR2^+^ ECs and FAP^+^ fibroblasts was visualized by immunofluorescence staining. Blue: DAPI, green: CD31, red: CXCR2, French gray: FAP. (B) Volcano plot of differentially expressed genes before and after co-culture of CAR-M2 with FAP^+^ fibroblasts. (C) Representative Masson and Sirius red staining images of mice kidney tissue sections following the injection of PBS into the renal subcapsule (Sham and UUO), CAR-M2-loaded HAMA-CS hydrogel injected into the renal subcapsule (CAR-M2), and PBS into the renal subcapsule of MMP2 inhibitor mice (MMP2 inhibitor). Detection at day 7. Scale bars, 100 μm. (D) Average collagen deposition density by Masson staining in (C), *n* = 5. (E) Average collagen deposition density (the percentage of positively stained area per HPF) by Sirius red staining in (C), *n* = 5. (F) Re-clustering of endothelial cells from human kidney single-cell sequencing data based on regulon activity, t-SNE (t-distributed stochastic neighbor embedding) displayed by cell type (left), and kidney condition (right). (G) SCENIC analysis of cell stability in clusters derived from regulon activity-based dimensionality reduction. (H) Ranked plot of transcription factor activity in CXCR2^+^ ECs. (I) Differential expression of the *Rxra* gene among groups in mouse single-cell transcriptome samples. (J) Changes in the endothelial transcription factor RXRA after CAR-M2 treatment and in MMP2 inhibitor-treated mice. Blue: DAPI, red: CD31, green: Rxra, yellow indicates the co-labeling of CD31 with Rxra, Scale bars, 25 μm. Arrowheads indicate the double positive cells. (K) Statistical analysis of the number of CD31^+^ Rxra^+^ cells in (J), *n* = 5. (L) Cell co-culture protocol to verify that CAR-M2 activates the transcription factor Rxra by secreted MMP2. (M) After cell co-culture using protocol in (L), the lentiviral constructs Cxcr2^+^ ECs were collected to detect the changes in their Rxra and Cxcr2 expression levels. (N) Average collagen deposition density by Masson staining, mice kidney tissue sections following the injection of PBS into the renal subcapsule (Control), CAR-M2-loaded HAMA-CS hydrogel injected into the renal subcapsule (CAR-M2), and PBS into the renal subcapsule of Rxra agonist and inhibitor mice (Rxra agonist and Rxra inhibitor). Detection at day 14. *n* = 5. (O) Average collagen deposition density by Sirius red staining, mice kidney tissue sections following the injection of PBS into the renal subcapsule (Control), CAR-M2-loaded HAMA-CS hydrogel injected into the renal subcapsule (CAR-M2), and PBS into the renal subcapsule of Rxra agonist and inhibitor mice (Rxra agonist and Rxra inhibitor). Detection at day 14. *n* = 5. (P) Mitochondrial autophagy-related gene score in human normal and CKD groups. (Q) Apoptotic-related gene score in human normal and CKD groups. (R) Mitochondrial autophagy-related gene score in mice Sham, UUO, and CAR-M2 groups. (S) Apoptotic-related gene score in mice Sham, UUO, and CAR-M2 groups. (T) The changes in the expression levels of autophagy-related protein PINK1 and apoptosis-related protein active caspase-3. (U) The cell apoptosis rate after the aforementioned (T) co-culture was detected by flow cytometry, and the proportion of Annexin-APC positive cells was counted as the cell apoptosis rate. The detection time was 6 h after co-culture. *n* = 3. ∗*p* < 0.05, ∗∗*p* < 0.01, ∗∗∗*p* < 0.001; ns, not significant (one-way ANOVA). See also Figure S7.

Position relationship analysis identified Cxcr2^+^ ECs located in the peritubular capillary endothelium adjacent to interstitial FAP^+^ fibroblasts (Figure 7A). This distance, less than 100 μm, offers an accessible range for further regulation of Cxcr2^+^ ECs following CAR-M2 targeting to FAP^+^ fibroblasts.^54^ Next, CAR-M2 before and after 24 h co-culture with FAP^+^ fibroblasts was sorted for bulk RNA sequencing and analyzed for differential genes. The analysis results showed that CAR-M2 significantly upregulated MMP2 after co-culture with FAP^+^ fibroblasts (Figures 7B and S7A–S7C) and MMP2 was involved in the remission of fibrosis through collagen degradation.^55^ The MMP2 inhibition in UUO mice revealed an increase in fibrosis compared to the CAR-M2-treated group (Figures 7C–7E). The above results suggested that MMP2 may be a key factor in the regulation of renal fibrosis by CAR-M2.

The transcription factor changes of Cxcr2^+^ ECs were further analyzed, and the transcription factor *Rxra* of Cxcr2^+^ ECs was found to be specifically activated in CKD (Figures 7F–7H and S7D–S7F). This is consistent with RXRA inhibition in kidney disease situations and activation during kidney recovery.^56^ Combined with mouse scRNA-seq analysis, we confirmed that *Rxra* was indeed upregulated in the CAR-M2 group (Figure 7I) and that the endothelial transcription factor Rxra is downregulated in mice where MMP2 was repressed (Figures 7J and 7K). The addition of MMP2 and Rxra inhibitors in the cell co-culture system of CAR-M2 (activated by FAP target protein)-regulated Cxcr2^+^ ECs also confirmed that CAR-M2 may regulate *Rxra* of Cxcr2^+^ ECs through secretion of MMP2 (Figures 7L and 7M). In the UUO mouse model, the inhibition of Rxra resulted in higher collagen deposition compared to the activation of Rxra. However, the activation of Rxra in the UUO group still showed a higher collagen deposition than in the CAR-M2 group, which suggested an important role of phagocytosis of FAP^+^ fibroblasts in relieving fibrosis (Figures 7N and 7O). The CKD did not lead to an increase in mitochondrial autophagy (Figure 7P) and apoptosis (Figure 7Q) in Cxcr2^+^ ECs. In contrast, activation of Rxra in Cxcr2^+^ ECs using CAR-M2 treatment induced an increase in mitochondrial autophagy (Figures 7R and 7T), which further resulted in apoptosis (Figures 7S and 7U) of Cxcr2^+^ ECs. The above results indicate that CAR-M2 activates the transcription factor Rxra in Cxcr2^+^ ECs through Mmp2, inducing apoptotic of pro-fibrotic Cxcr2^+^ ECs, thereby achieving a mitigating effect on renal fibrosis.

## Discussion

The current study shows that renal fibrosis is not evenly distributed throughout the renal parenchyma, but starts locally, forming a unique and aggregated niche (fibrogenic niche). Fibrogenic niche formation is associated with a series of complex and highly dynamic cellular events, including renal tubular cell injury, inflammatory cell infiltration, myofibroblast activation, tubular atrophy, and microvascular rarefaction.^25^ Thus, targeting and disrupting the fibrotic niche may be an effective strategy to cure renal fibrosis. The dual functionality of CAR-M2 in both targeting and regulating the components of the fibrogenic niche offers a promising strategy to prevent the onset and progression of renal fibrosis.

Renal fibrosis involves the dynamic interactions of various cellular types, including immune cells, stromal cells, and ECs, among others. Macrophages play important roles in regulating the progression and regression of fibrosis.^57^ M2 macrophages have a broad range of functions, including the inhibition of inflammation and the promotion of tissue repair. Macrophages influence fibrosis by regulating EC subsets that exhibit a profibrotic molecular phenotype. This endothelial subset in turn further recruits profibrotic immune cells or stimulates fibroblast activation to accelerate the progression of fibrosis.^7^^,^^26^ In this study, we revealed that the interaction between M2 macrophages and ECs plays a pivotal role in promoting the regeneration of the renal microvascular network and in promoting renal regeneration after fibrosis. We further identified Cxcr2^+^ ECs associated with renal fibrosis, which promote the process of fibrosis through the secretion of renal fibrosis-related protein, Lcn2. The infiltration of Cxcr2^+^ ECs and expression of Lcn2 were increased in human CKD. The transcriptomic analysis of mouse fibrotic samples and human CKD samples explores how CAR-M2 regulates profibrotic Cxcr2^+^ ECs. Initially, we discovered Cxcr2^+^ ECs were predominantly situated within the renal peritubular capillaries and with close physical proximity to FAP^+^ fibroblasts. This positioning facilitates the subsequent regulation of Cxcr2^+^ ECs following the CAR-M2 targeting of FAP^+^ fibroblasts. The regulation of specific ECs and organ vascularization provides a perspective for the treatment of organ fibrosis.

Precision drug delivery remains a big challenge in the treatment of parenchymal organs. Resection of the fibrotic kidney tissue is frequently undesirable, due to the whole renal interstitial distribution properties of renal fibrosis. *In situ* delivery may be a more specific and effective approach to match the anatomy of kidney and the distribution characteristics of fibrogenic niche. Here, we propose that the administration of renal subcapsular injection can be clinically applied with ultrasound guidance. The CAR-iMAC product derived from induced pluripotent stem cells (iPSCs) for tumor treatment has been approved to enter clinical trials.^58^ iPSC-based cell therapy offers significant advantages, including the capability for pre-manufacture and cryopreservation, making it highly promising for personalized cell therapy applications.^59^ Inspired by the allogeneic universal CAR-T therapy,^60^ using gene editing technology to further optimize hPSC-CAR-M2, reduce its immunogenicity, and improve clinical efficacy makes it possible for iPSC-derived CAR-M2 to become an off-the-shelf treatment option for organ fibrosis. This study provides a tangible integrated solution for this vision by combining iPSC-derived CAR-M2 cells with an injectable HAMA-CS hydrogel. This platform directly addresses the dual bottlenecks of scalable production and precise local delivery, forming a viable “off-the-shelf” strategy that can be pre-manufactured, stored, and deployed on demand.

### Limitations of the study

This study concentrates on the *in situ* administration of CAR-M2 for the treatment of renal fibrosis, utilizing clinical CKD samples for analysis. During the molding and treatment process of the animals, we utilized renal subcapsular injection following open abdominal surgery. This method can be adapted for ultrasound-guided renal subcapsule injection in future clinical applications, thereby enabling minimally invasive treatment. Further studies were required for the clinical application of CAR-M2 and minimally invasive delivery strategies. Furthermore, we identified Cxcr2^+^ ECs, which are closely associated with renal fibrosis. It remains unclear, however, if this particular cell population is uniquely present in renal fibrosis or if it can serve as a detection marker for organ fibrosis, including lung and liver fibrosis, among others. Future research is required to explore these matters, which are crucial for the advancement of CAR-M2 therapies in fibrotic diseases and their potential clinical translation.

## Resource availability

### Lead contact

Requests for further information and resources should be directed to and will be fulfilled by the lead contact, Wen Zeng (zengw0105@tmmu.edu.cn).

### Materials availability

The CAR-M2 lentiviral sequence generated in this study is shown in Table S2 and can be obtained from the lead contact. Mouse lines (Cxcr2-DreERT2 and Cdh5-CreERT2, Cxcr2-DreERT2, and Rosa-LSL-RSR-tdTomato-DTR) generated in this study are available for sharing, although the requesting party may need to cover the associated processing and transportation costs. All reagents generated in this study are available from the lead contact with a completed materials transfer agreement.

### Data and code availability

• scRNA-seq data have been deposited in the GEO database and are publicly available as of the date of publication. The accession numbers are listed in the key resources table. All data are available upon request to the lead contact, Wen Zeng (zengw0105@tmmu.edu.cn).
• No custom code was generated in this study.
• Any additional information required to reanalyze the data reported in this paper is available from the lead contact upon request.

## Acknowledgments

This study was supported by grants from the National Key Research and Development Plan Young Scientists Program Grant (2021YFA1102100), the 10.13039/100014717National Science Fund for Excellent Young Scholars (31771057), the 10.13039/501100019531Science Fund for Distinguished Young Scholars of Chongqing Municipality (cstc2020jcyj-jqX0023), and the high-level talent training program of Army Military Medical University (2022XRC01). We thank the Department of Pathology at the Eighth Medical Center of Chinese PLA General Hospital for providing human kidney pathological tissue sections and immunohistochemical staining support.

## Author contributions

W. Zeng and W. Zhao conceived and designed the study. W. Zhao, Xingli Zhao, and M.L. contributed to the *in vitro* cell experiments. Xin Zhao, L.C., and X.L. performed the experiments, analyzed the data. H.T., S.Z., Q.Z., Y.W., J.Y., X.L., and J.L. contributed to the animal studies, tissue processing, and staining experiments. Y.S., D.L., and L.L. analyzed and interpreted the data. Writing – original draft was done by W. Zeng, W. Zhao, X.Z., and H.T. Mechanism discussion and exploration were done by W. Zeng, B.L., W. Zhao and Xin Zhou. Supervision was done by W.C., B.L., C.Z. and W. Zeng. All authors have given approval to the final version of the manuscript.

## Declaration of interests

The authors declare no competing interests.

## STAR★Methods

### Key resources table

**Table:** 

REAGENT or RESOURCE	SOURCE	IDENTIFIER
**Antibodies**		

PE anti-human CD14 antibody	Biolegend	Cat#301806; RRID:AB_314188
CD10 antibody (F-4) FITC	Santa Cruz	Cat#sc-46656 FITC; RRID:AB_626828
Anti-FAP antibody [RM1080]	Abcam	Cat#ab314456; RRID:AB_3097779
CD31 (PECAM-1) (89C2) Mouse mAb	CST	Cat#3528T; RRID:AB_2160882
Anti-CD31 antibody [RM1006]	Abcam	Cat#ab281583; RRID:AB_3096925
Anti-F4/80 antibody [EPR26545-166]	Abcam	Cat#ab300421; RRID:AB_2936298
Arginase-1 Monoclonal antibody	Proteintech	Cat#66129-1-Ig; RRID:AB_2881528
Anti-Periostin antibody [EPR20806]	Abcam	Cat#ab215199; RRID:AB_2924310
Anti-a-SMA/ACTA2 Antibody (Clone#1A4)	BOSTER	Cat#BM0002; RRID:AB_2811044
Anti-Collagen I Rabbit pAb	Servicebio	Cat#GB115707; RRID:AB_3676315
Anti-Collagen III Mouse mAb	ABclonal	Cat#A0817; RRID:AB_3661637
Ki-67 Polyclonal Antibody	Invitrogen	Cat#PA519462; RRID:AB_10981523
Anti-VE Cadherin antibody	Abcam	Cat#ab205336; RRID:AB_2891001
Anti-VE-cadherin antibody (immunohistochemistry)	ZSGB-BIO	Cat#ZA-0575; RRID:AB_3720803
Anti-CD34 antibody (immunohistochemistry)	ZSGB-BIO	Cat#ZM-0046; RRID:AB_2924244
Anti-CXCR2 antibody	Abcam	Cat#ab65968; RRID:AB_1139971
Anti-Lipocalin-2/NGAL antibody [EPR5084]	Abcam	Cat#ab125075; RRID:AB_10978084
Retinoid X Receptor alpha antibody	MedChemExpress	Cat#HY-P80309; RRID:AB_3102417
IL-4 Monoclonal antibody	Proteintech	Cat#66142-1-lg; RRID:AB_3084212
CD163 Polyclonal antibody	Proteintech	Cat#16646-1-AP; RRID:AB_2919836
Anti-iNOS Rabbit pAb	ABclonal	Cat#A14031; RRID:AB_2760886
Beta Actin Monoclonal antibody	Proteintech	Cat#66009-1-Ig; RRID:AB_2883475
GAPDH Monoclonal antibody	Proteintech	Cat#60004-1-Ig; RRID:AB_2920461
Alpha Tubulin Polyclonal antibody	Proteintech	Cat#14555-1-AP; RRID:AB_2212258
Alexa Fluor™ 488 goat anti-mouse IgG(H + L)	Invitrogen	Cat#A11001; RRID:AB_2534069
Alexa Fluor™ 568 goat anti-mouse IgG(H + L)	Invitrogen	Cat#A11004; RRID:AB_2534072
Alexa Fluor™ 568 goat anti-rabbit IgG(H + L)	Invitrogen	Cat#A11011; RRID:AB_143157
Alexa Fluor™ 647 goat anti-rabbit IgG(H + L)	Invitrogen	Cat#A21244; RRID:AB_2535812
Alexa Fluor™ 488 goat anti-rabbit IgG(H + L)	Invitrogen	Cat#A11008; RRID:AB_143165
HRP Labeled Goat Anti-Mouse IgG(H + L)	Beyotime	Cat#A0216; RRID:AB_2860575
HRP Labeled Goat Anti-Rabbit IgG(H + L)	Beyotime	Cat#A0208; RRID:AB_2892644
PINK1 Polyclonal antibody	Proteintech	Cat#23274-1-AP; RRID:AB_3084072
Active caspase-3 mouse antibody	Immunoway	Cat#YM3431; RRID:AB_3095515

**Chemicals, peptides, and recombinant proteins**		

DMEM	GIBCO	Cat#C11960500BT
RPMI 1640	GIBCO	Cat#C11875500BT
Primary renal fibroblasts culture medium system	icellbioscience	Cat#PriMed-iCell-003
Fetal bovine serum	GIBCO	Cat#10099-141C
Penicillin-Streptomycin	GIBCO	Cat#15140-122
Trypsin cell digestion fluid	ThermoFisher	Cat#25200056
DPBS	ThermoFisher	Cat#C14190500BT
DMSO	Solarbio	Cat#D8371
Tris	coolaber	Cat#CT11411
SDS	coolaber	Cat#CS9701
Triton X-100 Solution (10%, Sterile)	Beyotime	Cat#ST797
Tween 20	Beyotime	Cat#ST825
DiR membrane dye probe	Meilunbio	Cat#MB12482
Dio membrane dye probe	Meilunbio	Cat#MB4239-1
Western and IP cell lysates	Beyotime	Cat#P0013
Protease inhibitor Mixture (generic, 100×)	Beyotime	Cat#P1005
Protease phosphatase inhibitor mixture	Beyotime	Cat#P1045
RIPA lysate	Beyotime	Cat#P0013B
Loading buffer	Solarbio	Cat#D8371
PVDF membrane	Merck millipore	Cat#IPVH0010
Matrigel	Corning	Cat#354234
Goat serum	Beyotime	Cat#C0265
DAPI dye solution	Beyotime	Cat#C1005
Hoechst 33342	Mengbio	Cat#MBR007
4% paraformaldehyde tissue fixative	Biosharp	Cat#BL539A
Glycine	Solaribio	Cat#G8200
APS	Solaribio	Cat#A1010-500
10%SDS Solution	Solaribio	Cat#S1010
1M Tris-Hcl Buffer	Solaribio	Cat#T1020
1.5M Tris-Hcl Buffer	Solaribio	Cat#T1010
OCT frozen section embedding agent	SAKURA	Cat#4583
Methylprenylated hyaluronic acid	EFL	Cat#EFL-HAMA-150K
Luciferase substrate	Dowobio	Cat#DW3113
Microfil	Flow tech Inc	Cat#MV-117
DNase I	Roche	Cat#10104159001
Recombinant Human Flt3-Ligand	PeproTech	Cat#300-19
Recombinant Human GM-CSF	PeproTech	Cat#300-03
Recombinant Human M-CSF	PeproTech	Cat#300-25
Recombinant Human TGF-β1	PeproTech	Cat#100-21
SB265610	MACKLIN	Cat#S921626
ZINC00640089	MedChemExpress	Cat#667880-11-7
GM6001	aladdin	Cat#G274767
Recombinant human MMP2 protein	Solarbio	Cat#P02344
ANG 193109	MedChemExpress	Cat#HY-U00449
LG10268	MedChemExpress	Cat#HY-15340
Corn oil	MedChemExpress	Cat#HY-Y1888

**Critical commercial assays**		

Mouse IL-4 ELISA Kit	Beyotime	Cat#PI612
Mouse IL-10 ELISA Kit	Beyotime	Cat#PI522
Mouse IFN-γ ELISA Kit	Beyotime	Cat#PI508
Modified Sirius red staining kit	Solarbio	Cat#G1472
Modified Masson staining kit	Solarbio	Cat#G1346
BCA protein concentration determination kit	Beyotime	Cat#P0012
TSA Fluorescence Triple Staining Kit	ABclonal	Cat#RK05903
Cell Counting Kit-8	Beyotime	Cat#C0039
Annexin V-APC / PI Apoptosis Detection Kit	Elabscience	Cat#E-CK-A217
10× Genomics Chromium Genetic Analyzer	10× Genomics	RRID:SCR_019326
Biotinylated Human FAP Protein, His,Avitag™ (MALS verified)	ACROBiosystems	Cat#FAP-H82Q6
Spherotech Streptavidin Polystyrene Particles, 0.5%w/v, 5.0–5.9 μm	Spherotech	Cat#SVP-50-5
Alexa Fluor 488 NHS ESTER	Invitrogen	Cat#A20000
Agilent Bioanalyzer	Agilent	RRID:SCR_018043

**Biological samples**		

Human kidney tissue slice	Department of Pathology at the Eighth Medical Center of Chinese PLA General Hospital	N/A

**Deposited data**		

scRNA-seq data of mice	This paper	GEO: GSE252943 (https://www.ncbi.nlm.nih.gov/geo/query/acc.cgi?acc=GSE252943)
scRNA-seq data of human	Abedini et al.^53^	GEO: GSE211785 (https://www.ncbi.nlm.nih.gov/geo/query/acc.cgi?acc=GSE211785)
Bulk-RNA sequencing data	This paper	GEO: GSE282078 (https://www.ncbi.nlm.nih.gov/geo/query/acc.cgi?acc=GSE282078)

**Experimental models: Cell lines**		

Human Umbilical Venous Endothelial Cells (HUVEC)	ATCC	Cat#bio-81814
Primary human renal fibroblasts	icellbioscience	Cat#HUM-iCell-u013
THP-1	ATCC	Cat#bio-73174
Mouse bone marrow-derived macrophages (BMDMs)	This paper	N/A
hPSC-M	Jun Shen lab (State key laboratory of Experimental Hematology)^61^	N/A

**Experimental models: Organisms/strains**		

Mouse: balb/C	Shanghai Model Organisms Center	Cat#SM-003
Mouse: Rosa-LSL-RSR-tdTomato-DTR	Shanghai Model Organisms Center	N/A
Mouse: Cxcr2-DreERT2	This paper	N/A
Mouse: Cdh5-CreERT2	Shanghai Model Organisms Center	Cat#NM-KI-200173
Mouse: Cdh5-CreERT2; Cxcr2-DreERT2; Rosa-LSL-RSR-tdTomato-DTR	This paper	N/A

**Software and algorithms**		

ImageJ	https://imagej-nihgov.gate2.inist.fr/ij/	N/A
FlowJo	https://www.flowjo.com	10.8.1
Prism Graphpad	https://www.graphpad.com/features	Version10
Cell Ranger	https://10xgenomics.com/	7.0.1
R	https://www.r-project.org/	4.1.2
Seurat	https://satijalab.org/seurat	5.0.1
Harmony	https://github.com/immunogenomics/harmony	0.1.1
msigdbr	https://github.com/igordot/msigdbr	7.5.1
CellChat	https://github.com/sqjin/CellChat	1.6.1
SCENIC	https://github.com/aertslab/SCENIC	1.3.1
devtools	https://CRAN.R-project.org/package=devtools	2.4.5
ComplexHeatmap	N/A	2.9.3
ggpubr	https://CRAN.R-project.org/package=ggpubr	0.6.0
tidyverse	http://www.tidyverse.org	1.3.2
ClusterProfiler	https://bioconductor.org/packages/release/bioc/html/clusterProfiler.html	3.14.3
pheatmap	https://cran.r-project.org/web/packages/pheatmap	CRAN
Monocle	https://cran.r-project.org/web/packages/monocle	2.32.0

### Experimental model and study participant details

#### Cell lines and cell culture

The human monocytic cell line THP-1 and the human umbilical vein endothelial cell line HUVEC were obtained from the American Type Culture Collection (ATCC). Primary human renal fibroblasts (HUM-iCell-u013) were purchased from iCell Bioscience Inc. (Shanghai, China). All cell lines were authenticated by the vendors (ATCC provides STR profiling reports; iCell provides species identification by PCR). All cell lines were routinely tested for mycoplasma contamination using a PCR-based detection kit (Beyotime, China) and were consistently negative before experiments.

THP-1 cells were cultured in RPMI 1640 medium (Gibco) supplemented with 10% fetal bovine serum (FBS, Gibco) and 1% penicillin-streptomycin (Beyotime). HUVEC cells were cultured in the same complete RPMI 1640 medium. Primary renal fibroblasts were cultured using the specified fibroblast growth medium system (PriMed-iCell-003, iCell Bioscience). All cells were maintained at 37°C in a humidified incubator with 5% CO_2_.

BMDMs were extracted from 6 to 8 weeks balb/C mice according to a previously published protocol, with slight modifications.^62^ In short, mice were euthanized by appropriate methods to remove the excess blood, and the lower limbs were removed. A 23G needle was used to introduce it into the bone marrow to slowly collect it into a 50 mL centrifuge tube. After RBC removal, bone marrow was resuscitated in 1 mL bone marrow medium (20 ng mL^−1^ GM-CSF, 300-03, PeproTech) in DMEM with 10% FBS and filtered using a 100 mm cell strainer. The suspension (approximately 1 × 10^7^ live cells) was seeded into well plates containing medium, and a half volume of medium was added on day 4. Cells were washed with sterile PBS on day 6 and the medium was replaced with fresh DMEM 10% FBS medium on day 6.

#### Mice

Immunocompetent male BALB/c (Cat. SM-003) mice, aged 8 weeks, were acquired commercially.

(Shanghai Model Organisms Center, China). Cxcr2-DreERT2 and Rosa-LSL-RSR-tdTomato-DTR mice was purchased from Shanghai Model Organisms Center. After breeding with our laboratory’s Cdh5-CreERT2 mice, we have successfully developed and obtained Cdh5-CreERT2; Cxcr2-DreERT2; Rosa-LSL-RSR-tdTomato-DTR mice. The resulting offspring were genotyped and male Cxcr2-EC-Dre conditional knockout mice (8 weeks old) were used for experiments. All mice were housed under standard institutional conditions, and group sizes are specified in the figure legends. All protocols and procedures involving animal experiments were approved by the Experimental Animal Welfare Ethics Committee of the Third Military Medical University (approval number: AMUWEC20211795).

#### Human samples

Human kidney tissue samples and pathological sections were obtained from the Department of Pathology at the Eighth Medical Center of Chinese PLA General Hospital. The collection and use of all human tissues were conducted in accordance with relevant guidelines and regulations, and were approved by the Institutional Review Board (IRB) of the Eighth Medical Center of Chinese PLA General Hospital under protocol number 3092023101000312579. Written informed consent was obtained from all participants or their legal guardians for the use of tissue for research purposes. The samples consisted of two cohorts: (1) disease cohort: renal biopsy specimens from patients with focal segmental glomerulosclerosis (*n* = 8); (2) control cohort: histologically normal renal tissue obtained from the unaffected pole of nephrectomy specimens from patients undergoing surgery for small renal tumors (*n* = 5). A portion of each specimen was processed into formalin-fixed, paraffin-embedded (FFPE) blocks for histopathological evaluation and immunohistochemical staining; the remaining tissue was snap-frozen for subsequent molecular analyses. Clinical information including sex, race, ancestry, and ethnicity was not provided by the hospital and therefore could not be incorporated into downstream analyses, which is acknowledged as alimitation.

### Method details

#### Construction and characterization of CAR-M2

##### Construction of plasmid and lentiviruses

Lentiviruses were constructed using CAR constructs cloned into the third-generation pTRPE lentiviral backbone using standard molecular biology techniques. All CAR constructs were generated using the CD8 lead sequence (GGGGS) 3 linker, CD8 hinge, and CD8 transmembrane domain and expressed under the control of the EF1 α promoter with the red fluorescent protein Scarlet or green fluorescent protein GFP. The lentivirus was packaged in HEK293 cells. Cells were transduced with lentiviral MOI 25–30 unless otherwise indicated.

##### CAR transduction and M2 polarization

For the *in vitro* co-culture experiments,a total of 50 ng/mL phorbol 12-myristate 13-acetate (PMA, P1585, MERCK) was added to a human monocyte culture system (RPMI with 10% FBS) for 24 h to convert human monocytes into macrophages, and then exposed to 20 ng/mL recombinant interferon-γ (300-02, PeproTech) and 100 ng/mL lipopolysaccharide (L8880, Solarbio) in RPMI with 10% FBS to induce M1 for 48 h. After completing the lentiviral transduction with CAR-M2, the M2 phenotype was detected following activation of the FAP target protein for 48 h.

##### Inflammatory factor detection by enzyme-linked immunosorbent assay (ELISA)

Samples were divided into cell culture medium and blood samples. Cell culture medium samples were centrifuged at 3000 rpm and 4°C for 10 min, and immediately subjected to the detection of IL-4, IL-10, TNF- α after the supernatant was removed. As regards the blood samples, the collected whole blood was kept overnight in a 4°C refrigerator and then centrifuged for 10 min at 3000 rpm and subjected to the detection of IL-4, IL-10, TNF- α immediately after supernatant removal. Briefly, samples or standards were seeded into a 96-well plate and incubated for 90 min at 37°C. Then, the prepared biotin-labeled detection antibody was added, incubated at 37°C for 60 min, and the enzyme-streptavidin-horseradish peroxidase mixture (IL-4, IL-10, TNF- α, Beyotime, MIbio) was added. After 37°C incubation for 30 min, tetramethylbenzidine (TMB) solution was added to the wells until the desired color appeared, and then TMB stop solution was added. The absorbance of the sample was detected at 450 nm.

##### Polarization identification of M2

The transition of CAR-M2 to M2 phenotype was assessed by setting the group of transduced CAR-M2 and BMDM cells without transduction with lentivirus as the control group. After 24 h of treatment, the medium was replaced with fresh medium, which was changed every day. After transduction, the expression changes of M1 (iNOS, TNF-α, and CD86) and M2 (arginase-1, CD163, and IL-10) markers in M2 BMDMs were detected by qRT-PCR, flow cytometry, ELISA, and immunofluorescence. The changes in macrophage phenotype in HAMA-CS hydrogel were assessed by measuring the M2 marker CD206 (321104, Biolgend) by flow cytometry 7 days after transduction. The flow cytometry analysis was performed by removing the transduced M2 BMDMs from the culture plate and blocked using TruStain FcXPLUS Antibody (Biolgend), then stained with the fluorescent-conjugated antibodies for 30 min at 4°C.

##### Real-time RT-PCR

Total RNA extraction kit was used to extracted total RNA in this work (Tiangen Biotechnology Co., Ltd., Beijing, China). RNA concentration and purity were determined by nanodrop photometry (Thermo Scientific). The cDNA was synthesized using a FastKing RT kit (Tiangen Biotech). The primer sets for real-time PCR are provided in the Table S1.^63^ Real-time PCR was performed on a Real-time PCR Detection System (Bio-Rad) using SYBR Green PCR Mater Mixture reagent (1725121, BIO-RAD).

##### Assessment of macrophage viability after CAR lentivirus transduction

The viability of M2 macrophages after CAR lentivirus transduction was determined using the cell counting kit-238 (CCK-8) assay (CCK 8-BS350B, biosharp). A total of 10^5^ M2 BMDMs were seeded into 24-well culture plates and transduced with MOI 30 CAR lentivirus after 24 h. Twenty-four hours after transduction, the medium was replaced with fresh medium, and cells were further cultured for 192 h. After adding the CCK-8 solution to each well, cells were incubated for 2 h. Absorbance was measured at 450 nm.

#### Construction and characterization of HAMA-CS hydrogel loading CAR-M2

##### HAMA-CS hydrogel preparation

The HAMA hydrogel used in this work was EFL-HAMA-150k (EFL-HAMA-150K, EFL). Photoinitiator LAP was dissolved in 10 mL PBS in 0.25% (w/v) initiator standard solution. Then, the desired mass of HAMA was weighed and mixed with LAP initiator standard solution and stirred at 37°C for 30 min. Finally, the resulting solution was sterilized using a 0.22 μm sterile pillow filter and kept at 4°C protected from light. Glacial acetic acid (1%) was added and the pH was adjusted to approximately 5 by NaOH solution. Then 1 g chitosan powder was added in 50 mL of this solution and placed to stir for 2 h at room temperature. A clear 2% chitosan solution was then adjusted to a neutral pH using a NaOH solution, sterilized using a 0.22 μm sterile pillow filter and stored 4°C in the dark. In the configuration of the cell-loaded hydrogel, the HAMA solution, chitosan-solution, and the cell suspension solution were cross-linked for 15s.

##### Identification of live and dead cells

A total of 10^6^ CAR-M2 were seeded in HAMA-CS hydrogel and incubated at 37°C under 5% CO_2_. After cell growth and release, the culture medium was removed, and HAMA-CS was degraded. The cells were rinsed with sterile PBS and incubated with 2 μM calcein (AM) and 8 μm M propidium iodide (PI) for 45 min. Finally, the cells were Bead-based phagocytosis assay. Refer to the reported methods.12 Strepavidin-coated polystyrene microparticles (5.0–5.9-μm diameter) were sterilized for 20 min in 70% isopropanol.

#### *In vitro* CAR-M2 targeting and phagocytosis of human kidnet fibroblasts

##### Bead-based phagocytosis assay

Refer to the reported methods.^12^ Strepavidin-coated polystyrene microparticles (5.0–5.9-μm diameter) were sterilized for 20 min in 70% isopropanol.

Beads were spun down and resuspended in 0.1M sodium bicarbonate buffer (pH 8.5) and labeled with 10 μM Alexa Fluor 488 NHS ester (Thermo Fisher) for 30 min in the dark. Beads were incubated with Biotinylated proteins from ACROBiosystems for 1 h, washed and resuspended in PBS for use in experiments. CAR-M2 was co-culture with FAP+ beads at a 1:1 ratio for 6 h. The RFP^+^GFP^+^ cells in the RFP^+^ population are thought to be cells being engulfed by macrophages.

##### Phagocytosis experiments based on live-cell workstations

A microscopy-based phagocytic assay of CAR-M2 and FAP^+^ fibroblasts. Control or CAR expressing THP-1 cells expressing red fluorescent protein positive (mScarlet) were cultured at a density of 10^5^/well in a 48-well plate and differentiated with 50 ng/mL PMA in RPMI containing 10% FBS for 24 h. After differentiation, PMA was washed with culture medium, 10^5^ control or target GFP^+^ human renal fibroblasts were added and cultured at 37°C for 24 h with 1 h intervals for mRFP and GFP fluorescence imaging. The average number of phagocitic events in six random fields (20× field of view) per well in three wells was calculated. The effect of different E:T ratios on phagocytosis was assessed by co-culturing hPSC-CAR-M2 with TGF-β-induced FAP^+^ fibroblasts and then detecting with a live-cell microscopy.

#### *In vivo* experiments in mice

##### Animal experiment design

Ureter ligation (UUO) mouse model was performed as follows: in brief, mice were anesthetized by an intraperitoneal injection of 0.5% sodium pentobarbitone. The abdominal skin of the left kidney was cut open, the left kidney was exposed, the left ureter was tied with a silk thread, and the ureter was cut off. Mice were maintained at a constant body temperature of 37°C. The Sham group only underwent anesthesia and muscle incision, and the left renal ureter was separated.^64^ Immediately after ligation, treatment was performed in 7 groups (*n* = 5): i) Sham group (Sham); ii) untreated UUO group (UUO); iii) UUO group, then CAR-M2 solution injection through the tail vein (T/CAR-M2); iv) CAR-M2 solution injection in renal subcapsule after UUO (S/CAR-M2); v) CAR-M2 HAMA-CS hydrogel injection in renal subcapsule after UUO (S/CAR-M2@HC); vi) M2 HAMA-CS hydrogel injection in renal subcapsule after UUO (S/M2@HC); vii) HAMA-CS hydrogels loaded with CARM and IL 4 were injected in renal subcapsule after UUO (S/CARM+IL4@HC). Treatment efficacy was evaluated using healthy mice without any manipulation or treatment as a normal control group. Mice were sacrificed on day 7, 14, and 21 after the initial UUO surgery. Immediately after UUO surgery, a HAMA-CS hydrogel containing 5 × 10^5^ CAR-M2 was injected through the renal subcapsule or the tail vein, with cell suspension or blank PBS as the control experimental group. Kidney tissue and serum samples were collected at day 7,14, and 21 to detect the changes of creatinine and nitrogen levels in the injured kidney.

##### Quantification of mouse biochemical indicators

Fresh mouse blood samples were centrifuged for 10 min at 2500 rpm. Serum creatinine (Cr) levels and blood urea nitrogen (BUN) were measured using an automated biochemical analyzer (Chemray 240) according to the manufacturer’s instructions.

##### Fluorescence imaging *in vivo*

DiR membrane dye probe working solution (MB12482, Meilunbio, 1:200) was used to fluorescently label CAR-M2 for 1 h, and unbound probes were washed off with PBS for 3 times. Then, CAR-M2 were re-suspended in the HAMA-CS solution and injected under the renal subcapsule for photocuring. The mice were anesthetized at day 7,14 and 21 time points for skin preparation required for FRI (NEWTON 7.0 FT-100.VILBER). Filter parameters are set to 782 nm emission and 750 nm excitation.^65^

For the *in vivo* survival assay with luciferase assay, luciferase CAR-M2 was constructed by luciferase lentivirus transduction, and luciferase substrate (DW3113, Dowobio) was added before fluorescence imaging. The degradation of the HAMA-CS hydrogel was detected by mixing the same DIR dye as described above with the HAMA-CS hydrogel.

#### *In vitro* tissue and tissue section experiments

Hematoxylin-eosin (H&E) staining, Masson staining, and Sirius red staining.

The kidney tissue and other main organs (heart, liver, spleen, and lung) in each group were fixed with 4% formaldehyde and embedded in conventional paraffin. After dehydration using ethanol gradients, paraffin (4 μm) slices were cut using a microtome. The slides were subsequently stained and the main renal pathology, collagen deposition, and fibrosis were observed using a whole wave slide scanner (Olympus, VS 120).

Tissue immunohistochemistry and immunofluorescence staining.

Kidney tissues embedded in paraffin were used in this work. Subsequently, paraffin sections, gradient alcohol dehydration, and antigen repair were performed in succession. After blocking with 3% hydrogen peroxide solution or 3% bovine serum, sectioned tissues were incubated with primary antibodies overnight at 4°C. Sectioned tissues were washed with PBS, treated with fluorescent secondary antibodies, and incubated for 50 min at room temperature. Subsequently, the staining was developed using DAB, and DAPI was used to stain the nucleus. Finally, the sections were dehydrated, mounted, microscopy-examined, image-acquired, and analyzed.

Sections of other main organs (heart, liver, spleen, and lung) were mainly used to verify the specific expression of FAP in the fibrotic kidney, and immunofluorescence staining of FAP was used. The intensity of the specific immunohistochemical staining was measured using the Image Pro Plus software (Media Cybernetics, USA). Co-staining of the same source antibody was performed using a TSA Fluorescence Triple Staining Kit (RK05903, ABclonal).

Micro-computed tomography scanning.

Microfil angiographic contrast agent (Flow Tech Inc, MV-117) was injected into the left ventricle of mice at a dosage of 1.5–2 mL kg-1. The renal pedicle was clamped at the end of perfusion, the kidney was removed, and then fixed overnight with 4% paraformaldehyde at 4° C. The fixed kidney was scanned using a microcomputed tomography (μCT) scanner (Skyscan 176, Bruker micro CT) with 6 micron voxel resolution.^2^

Flow cytometry scRNA sequencing cell sorting.

Single cell suspension was stained in FACS buffer (PBS, 1% BSA, sc-46656, Santa Cruz biotechnology) antibody with fluorescent dye-labeled CD10 (0.05% sodium azide) and sorted with Aria sorp and cell sorter (BD Biosciences, USA). Cell debris was excluded by FSC and SSC parameters, cell adhesion by SSC or FSC W and H parameters, and dead cells by 7-AAD and calcein AM to select cells with good viability. The CD 10-positive cells were then sorted. CD10^+^ cells and CD10^−^ cells were separately sorted from kidney samples and mixed at a 1:2 ratio.

Establishment and sequencing of single cell libraries.

The 10× Genomics 3′ transcriptome uses short read long sequencing and microfluidic technology for simultaneous transcriptome expression profiling of 500-10,000 cells per sample. The cDNA digestion was first broken into fragments of approximately 200–300 bp, plus the traditional second-generation sequencing process of sequencing connector P5 and sequencing primer R1, and finally amplified by PCR to obtain the DNA library. Library sequencing was performed at high throughput using the two-end sequencing mode of the Illumina sequencing platform. At the Read 1 end, 16 bp of barcode information and 10 bp of UMI information were used to determine cells and expression quantification; at the Read 2 end, the cDNA fragment was used to identify the genes corresponding to the mRNA.

#### RNA-seq analysis

##### Single-cell sequencing data analysis

The 10× Genomics official analysis software Cell Ranger was used to perform data quality statistics on the raw data, which were compared to the reference genome. Subsequently, the single-cell transcriptomic data were further used for quality control, analysis, and data exploration using the R package Seurat (version 5.0.1).^3^

##### Cell annotation

The Seurat’s FindAllMarkers function was used to perform differential gene expression analysis on each cluster (or subpopulation) to identify genes specifically expressed in each cluster (or subpopulation). This was combined with known cell type marker genes from public databases such as CellMarker2.0 (http://bio-bigdata.hrbmu.edu.cn/CellMarker/index.html) and PanglaoDB (https://panglaodb.se/). Highly expressed genes in each cluster were compared with the reference marker gene list. If a group of genes in a particular cluster highly overlapped with specific cell type marker genes, that cluster could be annotated as that cell type. If the match between differentially expressed genes and marker genes was not obvious, the GSEA enrichment analysis tool was used to evaluate the enrichment of marker gene sets in different clusters, thereby annotating a possible cell type for each cluster.

##### Pseudotime analysis

Monocle (v2.32.0) was used to import Seurat object data and create a CellDataSet object. Differential genes between subpopulations were used as ordering genes to construct cell trajectories. The DDRTree dimensionality reduction technique was employed to project high-dimensional expression data into low-dimensional space. Based on the cells’ positions in the resulting tree, pseudotime values were calculated, representing the distance of cells along the tree to its root. Cells were then ordered according to their computed pseudotime values to reflect their positions in the biological process.

##### Gene set scoring evaluation

Specific gene sets were downloaded from the MsigDB website (http://software.broadinstitute.org/gsea/msigdb/index.jsp) and obtained through literature, with detailed information about the gene sets provided in the supplementary table. The selected gene sets were converted into a format suitable for the AddModuleScore function. The AddModuleScore function from the Seurat package was used to calculate the activity of each gene set in every cell. This function computes the average expression value of the gene set in each cell and compares it with randomly selected background gene sets, outputting a gene module score for each cell. Violin plots were used to display the differences in gene set activities across different cell populations.

##### Cell interaction analysis

The Seurat object data was normalized and both genes and cells were filtered to remove low-expression noise data. The CellChat software package (v1.6.1) was then used to construct a cellchat analysis object. In this study, the CellChatDB.mouse ligand-receptor interaction database was selected, along with all data containing “Secreted Signaling” and “Cell-Cell Contact”. By identifying active signaling pathways, we estimated the strength of these pathways and calculated the probability of signal transmission between different cell types.

##### Functional enrichment analysis

Differential genes were identified using the FindAllMarkers function with a logfc.threshold of 0.25, while keeping other parameters constant. Based on the sample species, the differentially expressed genes were organized into an input gene list using the mouse org.Mm.e.g.,.db package (v3.19.1) for mice and the human org.Hs.e.g.,.db package (v3.19.1) for humans. The clusterProfiler software package (v4.12.0) was then used to perform functional enrichment analysis on the differential genes, focusing on pathways including GO and KEGG, with other parameters set to default. Significance thresholds included *p* < 0.05 or FDR-adjusted *p* values during the enrichment process.

##### Transcription factor analysis

The analysis of transcription factors was conducted using the SCENIC software package (version 1.3.1). In brief, Using GENIE3 or GRNBoost software, gene regulatory networks were inferred from single-cell gene expression data through random forest or gradient boosting algorithms. RcisTarget software was further employed to analyze the enrichment of transcription factor binding sites (motifs) on target genes based on the TF-gene co-expression network obtained in the first step. The activity level of regulons in each cell was calculated using AUCell software. AUCell evaluates the activity status of each regulon based on gene expression levels, producing an activity matrix for subsequent cell state analysis and clustering.

##### Human public data analysis

We integrated recently published sequencing data on human kidneys (GSE211785). The original data analyzed by the authors were downloaded and subjected to uniform quality control standards. Endothelial cells were extracted for further dimensionality reduction and clustering analysis. In the fibrosis grading, based on the original data, fibrosis scores were divided into four categories: a score of 0 was classified as category 0, scores of 1–10 as category 1, scores of 11–20 as category 2, and scores above 21 as category 3.

##### STRING database interaction analysis

Using “LCN2” as the keyword, a data search and analysis were conducted in the STRING database (https://cn.string-db.org/). An interaction network graph (PPI network) was generated based on the input protein, with nodes representing proteins and edges indicating their interactions. Different colors of lines represent varying evidence strengths.

##### Bulk RNA-Sequencing and data analysis

Bulk RNA-sequencing was conducted in two groups: one consisting of CARM cells, and the other of CARM cells following phagocytosis of FAP^+^ fibroblasts, with three replicates per group. RNA extraction was performed using the RNeasy Mini Kit (QIAGEN) according to the manufacturer’s instructions. cDNA library sequencing was carried out on the Illumina NovaSeq X Plus platform by Gene *Denovo* Biotechnology Co., Ltd (Guangzhou, China). The quality of the libraries was assessed using an Agilent BioAnalyzer 2100 (Agilent), and library concentrations were measured using library quantification kits (Biolabs). FPKM (fragments per kilobase of transcript per million mapped reads) values were generated, and differential expression analysis between the groups was conducted using DESeq2 (Love et al., 2014). Briefly, reads were aligned to the Mus musculus UCSC mm10 gene annotation (covering 24,421 genes) using the STAR aligner (v2.3.1.s). Genes were considered statistically significantly differentially expressed based on a relaxed threshold (adjusted *p*-value <0.05). All RNA-seq analysis figures were generated using R (version 3.5.0) and R Studio (version 1.1.383) with custom scripts available upon request.

#### *In vitro* Cxcr2^+^ ECs cell function assay

##### Construction of Cxcr2^+^ ECs and Co-culture system

Cxcr2^+^ ECs were constructed by transfecting HUVECs with Cxcr2 overexpression lentivirus. Co-culture them with CAR-M2 culture supernatant or other conditioned media supplemented with agonists or inhibitors to detect the protein expression levels of Cxcr2^+^ ECs themselves or proteins related to their functions such as apoptosis. Co-culture them with human renal fibroblasts to detect their regulatory effects on fibrosis-related genes.

##### Immunoblotting

After co-culturing for 6 h, the ECs or Cxcr2^+^ ECs cells were washed twice with pre-cooled PBS, and 100 μL of RIPA lysis buffer (containing 1 mM PMSF and a protease inhibitor cocktail) was added to prepare protein samples. After SDS-PAGE electrophoresis, the proteins were transferred to a PVDF membrane, and antibodies were used to detect the expression levels of Cxcr2, Rxra, and apoptosis-related proteins. ImageJ software was used to analyze the band grayscale values.

##### Annexin V-APC/PI apoptosis detection

After co-culturing for 6 h, the ECs or Cxcr2^+^ ECs cells were collected. Resuspend cells with pre-cooled 1× Annexin V binding buffer to adjust the cell density to 1×10^6^ cells/mL. Take 100 μL of the cell suspension, add 5 μL of Annexin V-APC, and incubate in the dark at room temperature for 15 min. Add 5 μL of propidium iodide and incubate in the dark for another 5 min. After staining, immediately add 400 μL of Annexin V binding buffer, gently mix, and keep on ice in the dark for preservation. The sample should be analyzed within 1 h.

### Quantification and statistical analysis

Unless otherwise specified, quantitative data are presented as mean ± standard error of the mean (SEM). Statistical analyses were performed using GraphPad Prism software (version 10 for Windows). The specific tests applied are as follows: Unpaired, two-tailed Student’s *t* test was used for comparisons between two groups. For comparisons among three or more groups, one-way analysis of variance (ANOVA) was applied, followed by appropriate post-hoc tests for multiple comparisons. two-way ANOVA was employed for analyses involving two independent variables (e.g., treatment and time). A *p*-value of less than 0.05 was considered statistically significant, and significance levels are denoted as follows: ∗*p* < 0.05, ∗∗*p* < 0.01, ∗∗∗*p* < 0.001. Exact values of n (representing biological replicates, e.g., number of animals or independent experiments) and the results of statistical tests are provided in the corresponding figure legends.

## Supplemental information

Document S1. Figures S1–S7 and Tables S1 and S2

Document S2. Article plus supplemental information
