# Supplementary material for: CAR-M2 immunotherapy resolves renal fibrosis via revascularization and apoptosis of profibrotic Cxcr2+ endothelial cells
Source: Cell Rep Med. 2026 Mar 25;7(4):102698. doi: 10.1016/j.xcrm.2026.102698 (PMC13130657; doi:10.1016/j.xcrm.2026.102698)
Supplement: Document S1. Figures S1–S7 and Tables S1 and S2 [file mmc1.pdf]

Cell Reports Medicine, Volume *7*

Supplemental information

CAR-M2 immunotherapy resolves renal fibrosis via revascularization and apoptosis of profibrotic Cxcr2^{+} endothelial cells

Wenyan Zhao, Xin Zhou, Xingli Zhao, Hao Tian, Yang Su, Shanlan Zhao, Min Liu, Qiao Zhang, Lin Chen, Xiaochen Li, Di Liu, Junxuan Li, Lang Li, Yanhong Wang, Xingtong Li, Jin Yan, Wen Chen, Bing Liu, Chuhong Zhu, and Wen Zeng

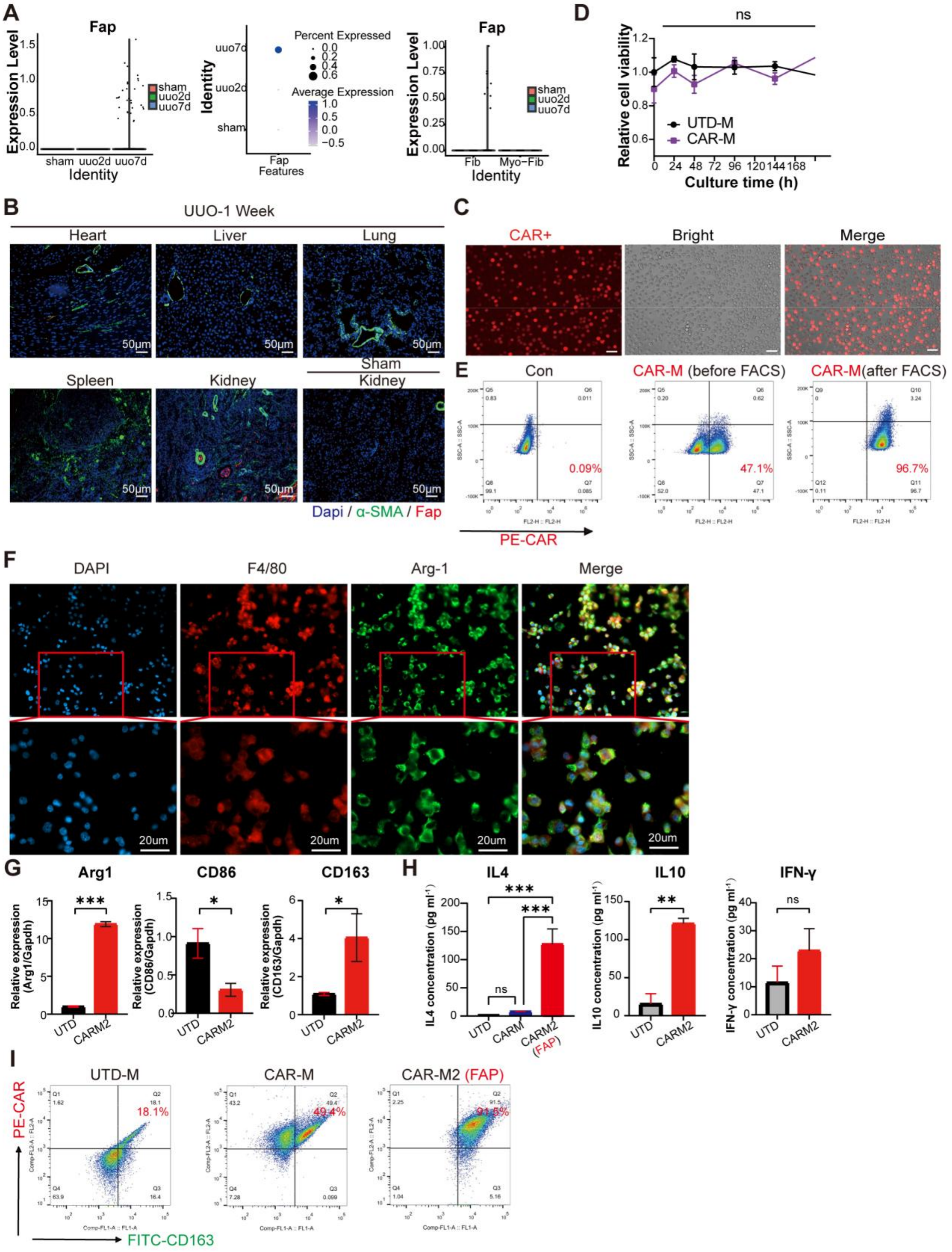

A
Fap
Expression Level
Identity
sham
uuo2d
uuo7d
Percent Expressed
Average Expression
Fap
Features
Fib
Myo−Fib
D
ns
Relative cell viability
UTD-M
CAR-M
Culture time (h)
B
UUO-1 Week
Heart
Liver
Lung
Spleen
Kidney
Sham
Kidney
50μm
Dapi / α-SMA / Fap
C
CAR+
Bright
Merge
E
Con
CAR-M (before FACS)
CAR-M(after FACS)
0.09%
47.1%
96.7%
PE-CAR
F
DAPI
F4/80
Arg-1
Merge
20um
G
Arg1
CD86
CD163
Relative expression (Arg1/Gapdh)
Relative expression (CD86/Gapdh)
Relative expression (CD163/Gapdh)
UTD
CARM2
H
IL4
IL10
IFN-γ
IL4 concentration (pg ml⁻¹)
IL10 concentration (pg ml⁻¹)
IFN-γ concentration (pg ml⁻¹)
UTD
CARM
CARM2 (FAP)
ns
I
UTD-M
CAR-M
CAR-M2 (FAP)
18.1%
49.4%
91.5%
PE-CAR
FITC-CD163

Figure S1. The CAR-M2 targeting Fap was constructed based on the specific and high expression of Fap in UUO mice, Related to Figure 1.

(A and B) Changes of Fap expression in UUO mouse kidney and other healthy organs analyzed by sc-RNA-seq (A) and immunofluorescence staining (B). Scale bar: 50 µm.

(C) Immunofluorescent images after transduction.

(D) Viability of anti-FAP-IL4-CAR-treated macrophages, as determined using CCK-8 assay (n=3).

(E) Representative FACS plot of CAR expression after transduced macrophages (right, mScarlet red fluorescence, CAR-M; left, UTD-M; n=3).

(F-I) Immunofluorescence staining at 72 h (F), qRT-PCR at 24 h (n=3) (G), ELISA at 72 h (n=3) (H), and flow cytometry at 72 h of CAR-M2 (I) demonstrated the successful programming of IL4-CAR-transduced macrophages M2 (with up-regulated Arg-1, CD163, IL4, and IL10, and down-regulated CD86).

$^*p < 0.05$, $^{**}p < 0.01$, $^{***}p < 0.001$ and not significant (ns) (two-tailed Student's *t* test).

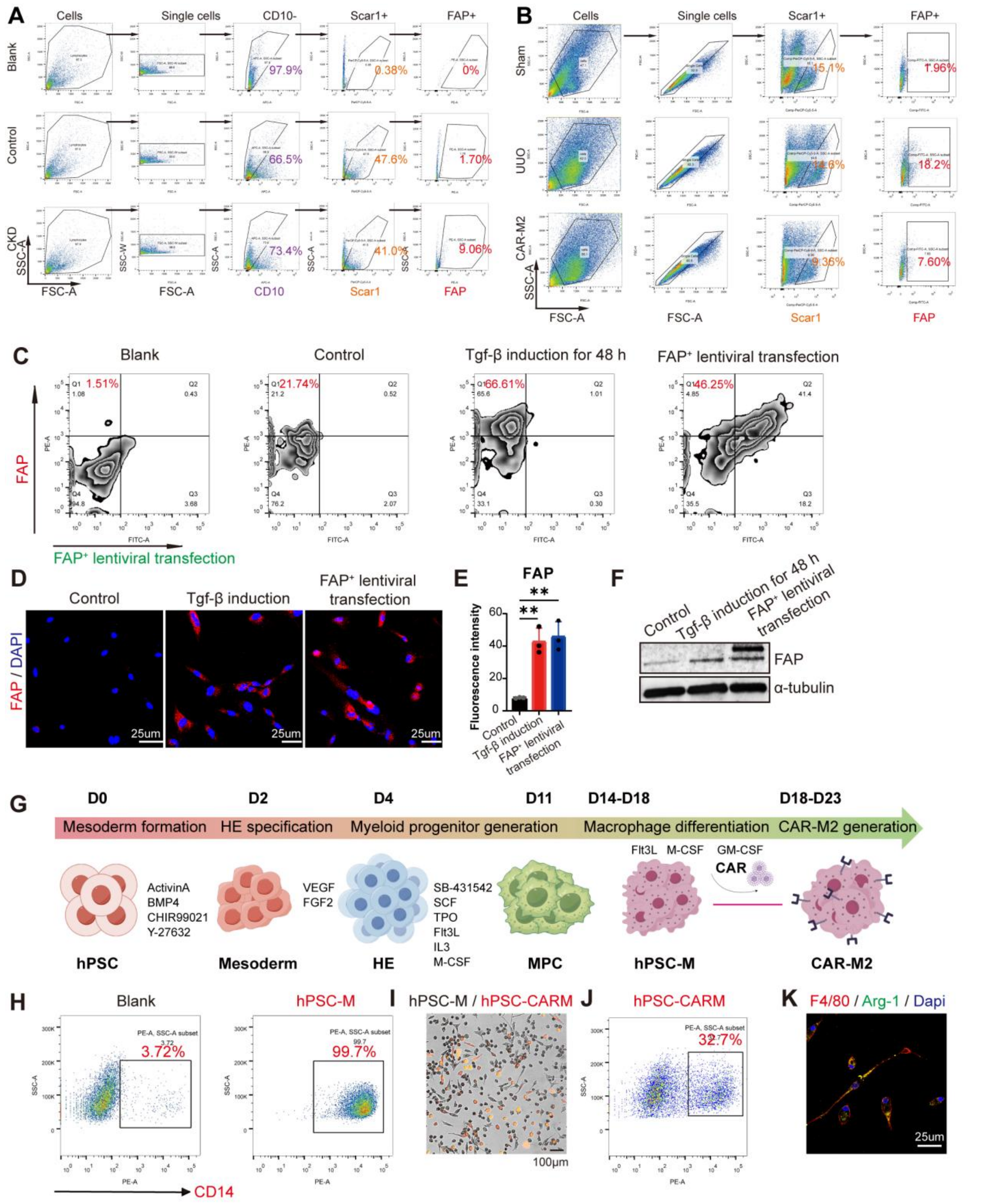

Figure S2. The FAP fibroblasts were specifically and highly expressed in the human CKD and UUO mice kidney, and the construction of fibrosis and phagocytosis models, Related to Figure 1.

(A) The expression of FAP on human CKD samples by flow cytometry. The FAP^{+}fibroblasts were identified as CD10^{-}Sca-1^{+}FAP^{+} cells, and FAP expression was quantified within this gated stromal population.

(B) The expression of FAP on Sham, UUO and CAR-M2 treatment samples by flow cytometry. The FAP^{+}fibroblasts were identified as Sca-1^{+}FAP^{+} cells, and FAP expression was quantified within this gated stromal population.

(C) Flow analysis of FAP expression in human renal fibroblasts induced by TGF-β and transduced by FAP lentiviral.

(D and E) Immunofluorescence staining and quantitative analysis of FAP. Nuclei were stained with

DAPI (blue), Scale bar: 25 μm.

(F) Western blot analysis of FAP expression in human renal fibroblasts induced by TGF-β and transduced by FAP lentiviral.

(G) Schematic diagram showing the protocol for generating CAR-M2 from hPSCs. HE, hemogenic endothelium; MPC, myeloid progenitor cells; hPSC-Ms, hPSC-derived macrophages.

(H) hPSCs were successfully induced as $CD14^{+}$ macrophages.

(I-K) CAR-M2 were successfully transduced and constructed.

**$p < 0.01$ (one-way ANOVA).

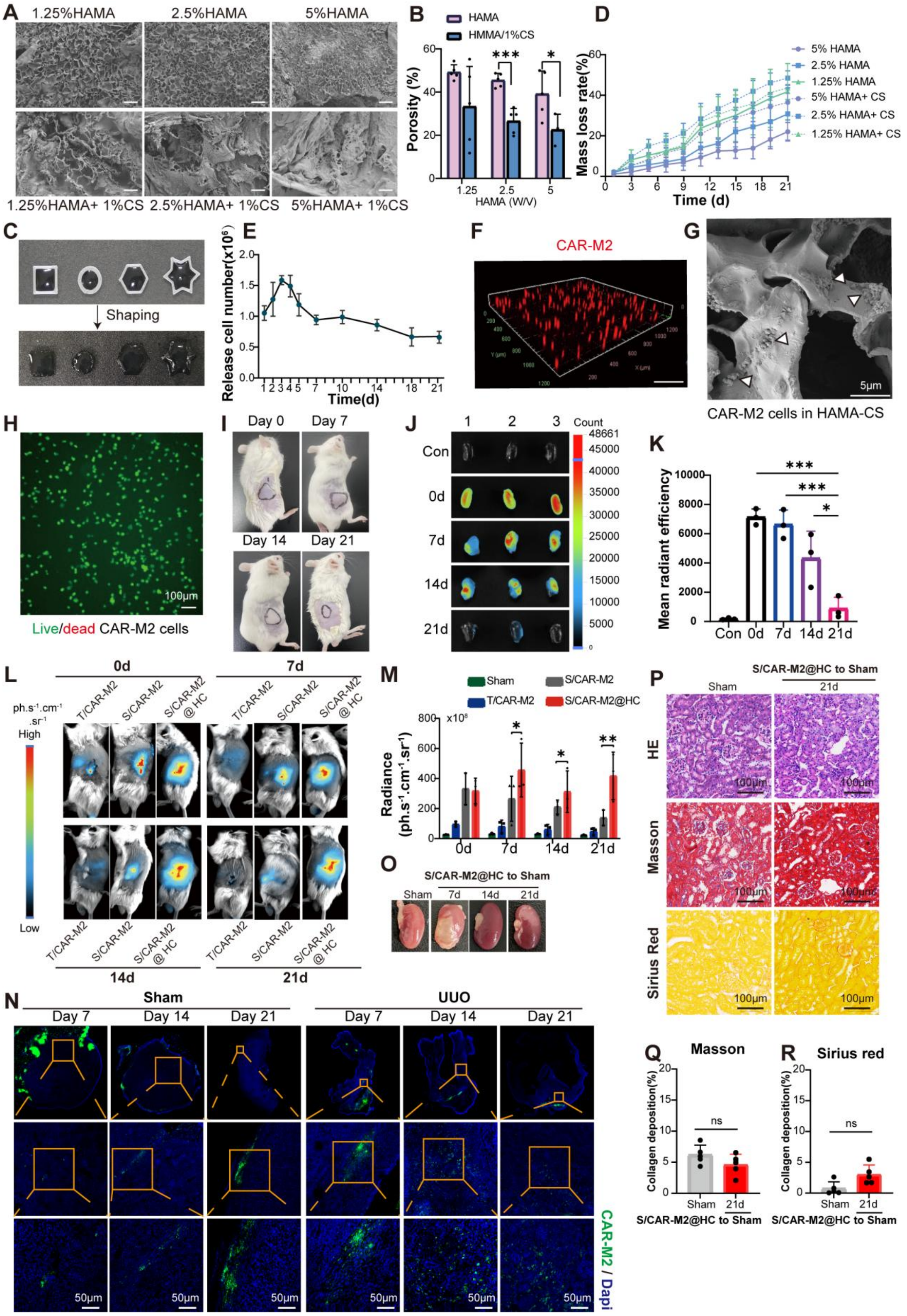

Figure S3. Characterization of the physical properties and biological performance of HAMA-CS loaded with CAR-M2, Related to Figure 2.

(A) Scanning electron microscopy imaging of lyophilized HAMA hydrogels and HAMA-CS hydrogels at different HAMA concentrations (1.25, 2.5, and 5%, w/v) (n=3). Scale bar: 100 μm.

(B) Porosity of the engineered hydrogels.
(C) Plasticity of the engineered hydrogels. Light curing time: 15 s.
(D) Degradation rate of the hydrogels (n=3).
(E) Release profile of CAR-M2 from the hydrogel *in vitro* (n=3). CAR-M2 density: 1×10^7.
(F) Confocal 3D stereoscopic imaging of CAR-M2 encapsulated in a 2.5% HAMA-1%CS hydrogel. Scale bar:, 200 μm.
(G) Scanning electron microscopy imaging of CAR-M2 (arrowheads mark position) in HAMA-CS hydrogel (n=3). Cell density: 2×10^6. Scale bar: 5 μm.
(H) Confocal imaging of live/dead assay of CAR-M2 released from the hydrogel at 7 d (n=3). Scale bar: 100 μm.
(I) Representative image of the subcutaneous degradation of the CAR-M2 loaded HAMA-CS hydrogel, n=5.
(J) The degradation of CAR-M2 loaded HAMA-CS hydrogel in kidney, n=3.
(K) Statistical analysis of the fluorescence intensity in (I).
(L) Extended and prolonged release of CAR-M2 loaded into HAMA-CS *in vivo*. Representative images of In vivo fluorescence imaging in UUO mice of CAR-M2 loaded hydrogels, n=5.
(M) Total radiant efficiency normalized useing manual measurements over the fluorescence region of interest and by the initial signal intensity, (n=5,Two-way ANOVA).
(N) Representative images of CAR-M2 released from the renal subcapsule HAMA-CS hydrogel into the fibrotic kidney compared to the Sham group, n=5.
(O) Kidney morphology in normal mice after treatment with CAR-M2.
(P) H&E, Masson, and Sirius Red staining of normal mice at 21 d after CAR-M2 treatment , n=5.
(Q and R) Statistical analysis of Masson and Sirius red staining.
*$p < 0.05$, **$p < 0.01$, ***$p < 0.001$ and not significant (ns) (two-tailed Student's t test, one-way ANOVA and Two-way ANOVA).

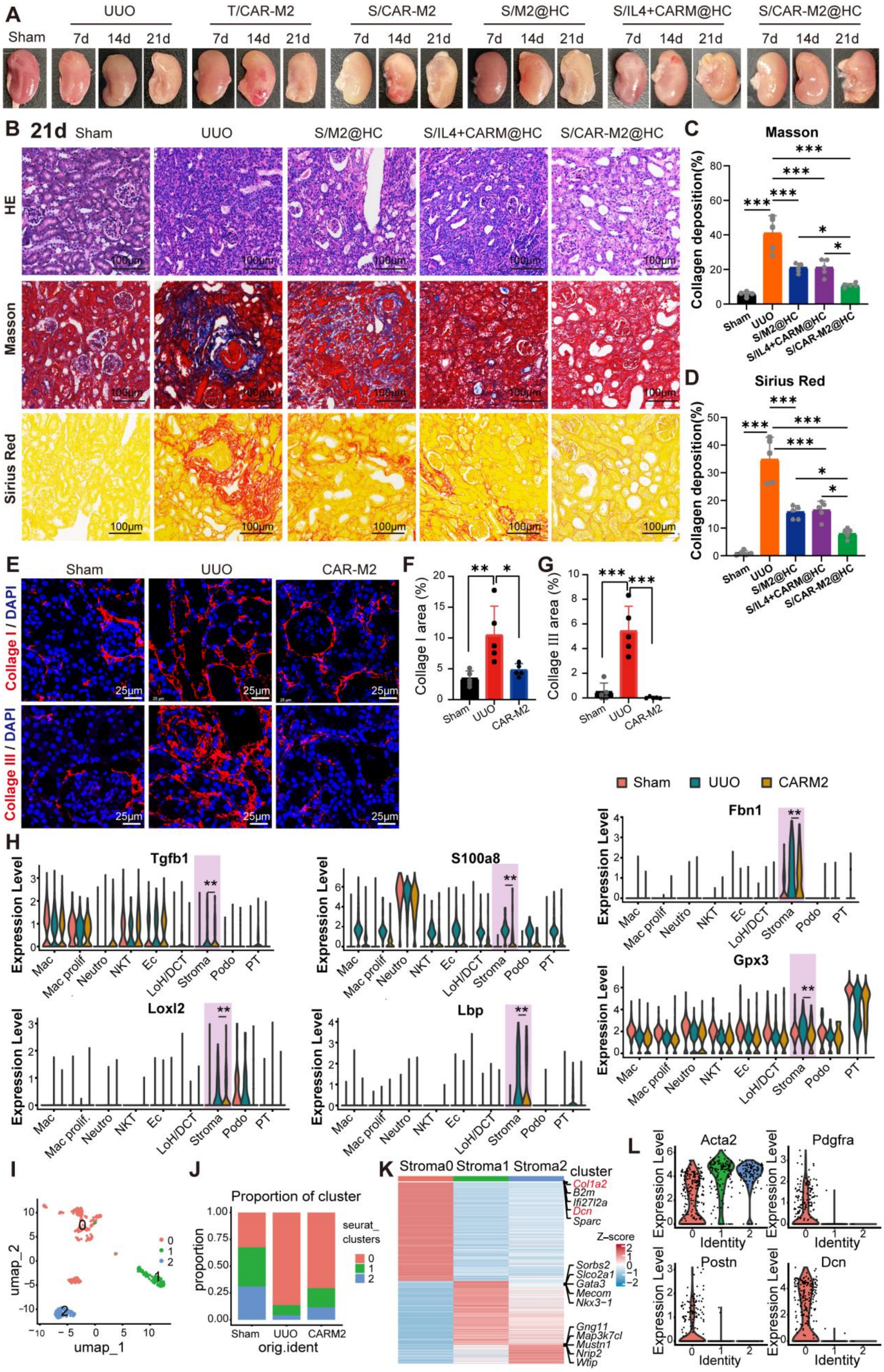

Figure S4. CAR-M2 decreased collagen deposition in fibrotic kidneys of mice, Related to Figure 2 and 3.

(A) Kidney morphology in UUO mice after treatment with the injection of PBS into the renal subcapsule (Sham and UUO), CAR-M2 solution by the tail vein injection (T/CAR-M2), CAR-M2 solutions by locally renal subcapsule injection (S/CAR-M2), M2 loaded hydrogel injected into the renal subcapsule (S/M2@HC), IL4 and CARM loaded hydrogel injected into the renal subcapsule (S/IL4+CARM@HC) and CAR-M2 loaded hydrogel injected into the renal subcapsule (S/CAR-M2@HC). Detection at day 7, 14 and 21.

(B) Representative H & E, Masson and Sirius Red staining images of mice kidney tissue sections following the injection of PBS into the renal subcapsule (sham and UUO), M2 loaded hydrogel injected into the renal subcapsule (S/M2@HC), IL4 and CARM loaded hydrogel injected into the renal subcapsule (S/IL4+CARM@HC) and CAR-M2 loaded hydrogel injected into the renal subcapsule (S/CAR-M2@HC). Detection at day 21. Scale bar: 100 μm.

(C) Average collagen deposition density [the percentage of positively stained area per high-power field (HPF)] by Masson staining in (B), n = 5.

(D) Average collagen deposition density [the percentage of positively stained area per high-power field (HPF)] by Sirius Red staining in (B), n = 5.

(E-G) Immunofluorescence staining and quantitative analysis of the collagen I and III protein levels. Scale bar: 25 μm.

(H) Expression of fibrosis-related gene expression in different groups and cell clusters. (one-way ANOVA).

(I) The UMAP plot displays the subclusters of stromal cells.

(J) Bar graph showing the proportion of each stromal cell cluster to the overall stromal cells.

(K) Heat map showing gene expression patterns in stromal cells. Each row represents one gene, and each column represents a cell cluster.

(L) The expression of representative marker genes (*Acta2*, *Pdgfra*, *Postn*, and *Dcn*) varies across different sub-clusters.

*p < 0.05, **p < 0.01 and ***p < 0.001 (two-tailed Student's t test and one-way ANOVA).

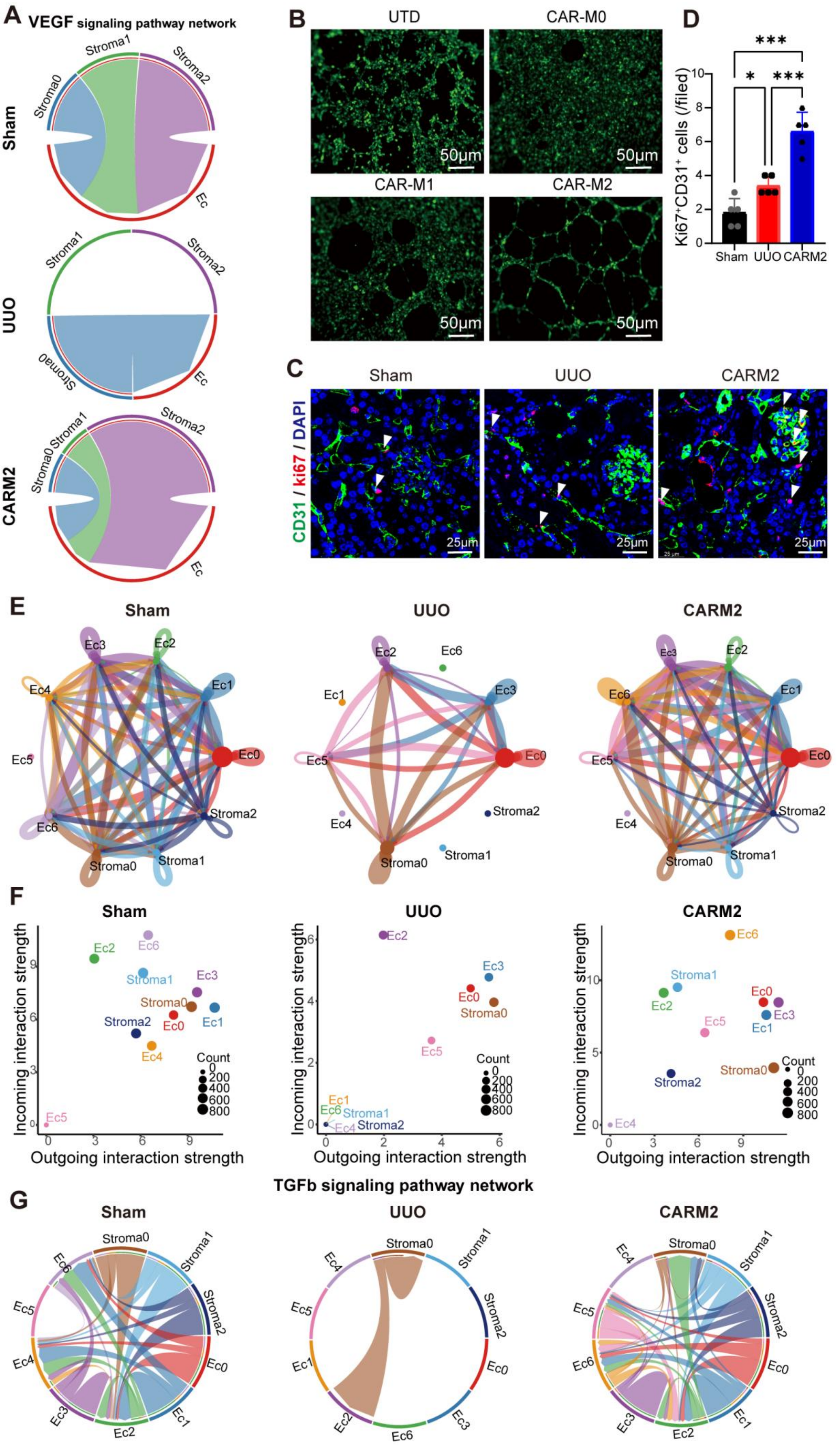

A VEGF signaling pathway network
Sham
UUO
CARM2
Stroma0
Stroma1
Stroma2
Ec
B
UTD
CAR-M0
CAR-M1
CAR-M2
50μm
D
Ki67+CD31+ cells (/filed)
Sham UUO CARM2
C
Sham
UUO
CARM2
CD31 / ki67 / DAPI
25μm
E
F
Incoming interaction strength
Outgoing interaction strength
Count
G
TGFb signaling pathway network

Figure S5. CAR-M2 modulate angiogenesis and the interactions between EC6 and renal fibrosis, Related to Figure 4 and 5.

(A) The changes in VEGF interaction signal among three sub-clusters of stromal cells and endothelial cells across the three groups.

(B) *In vitro* tube formation experiments demonstrated that CAR-M2 promote endothelial tube formation.

(C and D) An immunofluorescence co-staining of CD31 with ki67 demonstrated that renal subcapsule injection treatment with CAR-M2 loaded hydrogel promotes endothelial cell proliferation. Scale bar: 25 μm. n=3.

(E) General regulatory networks of endothelial sub-clusters and stromal cell sub-clusters in Sham, UUO and CAR-M2 treatment groups.

(F) The changes in outgoing and incoming signals of endothelial sub-clusters and stromal cell sub-clusters in Sham, UUO and CAR-M2 treatment groups.

(G) Regulatory networks of TGF-β signaling pathway in sham, UUO and CAR-M2 treatment groups.

*$p < 0.05$ and ***$p < 0.001$ (one-way ANOVA).

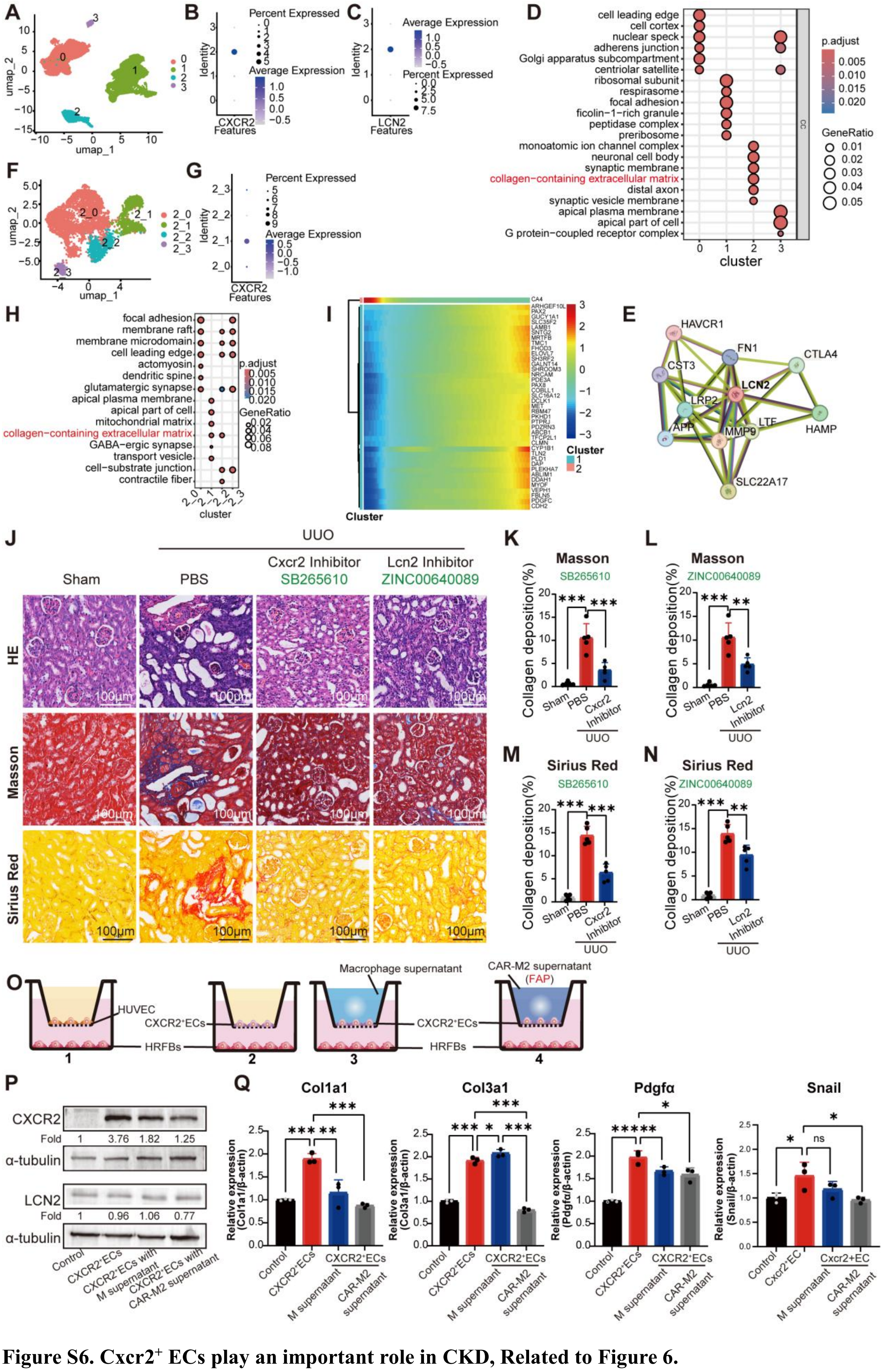

Figure S6. Cxcr2⁺ ECs play an important role in CKD, Related to Figure 6.

(A) UMAP plot showing re-dimensioned clustering of all endothelial cell sub-clusters in human kidney single-cell sequencing data.

(B) Expression differences of *CXCR2* among different clusters.
(C) Expression differences of *LCN2* among different clusters.
(D) GO-CC enrichment of differential genes among different clusters.
(E) STRING-based analysis of potential LCN2-binding molecules in human kidney.
(F) UAMP plot showing further dimension reduction clustering of population 2 for all endothelium with high CXCR2 expression.
(G) Expression differences of CXCR2 in subclusters of the cluster 2.
(H) GO functional enrichment of differential genes among subclusters of cluster 2.
(I) Heat map of expression changes of related genes during the pseudotime trajectory.
(J) Representative H & E, Masson and Sirius Red staining images of mice kidney tissue sections following the injection of PBS into the renal subcapsule (Sham and UUO), and PBS into the renal subcapsule of Cxcr2 and Lcn2 inhibitor mice (Cxcr2 inhibitor and Lcn2 inhibitor). Detection at day 14. Scale bar: 100 μm.
(K-N) Average collagen deposition density [the percentage of positively stained area per high-power field (HPF)] by Masson and Sirius Red staining in (J), n = 5.
(O) Cell co-culture scheme verified that CAR-M2 relieved fibrosis by regulating Lcn2 expression of Cxcr2^{+} ECs.
(P) After cell co-culture using scheme in (O), the lentiviral constructs Cxcr2^{+} ECs were collected for western blot to detect the changes in Cxcr2 and Lcn2 expression levels.
(Q) After cell co-culture using scheme in (O), the human renal fibroblasts (HRFBs) were collected for qPCR to detect the changes in their expression levels of the genes associated with fibrosis.
*$p < 0.05$, **$p < 0.01$, ***$p < 0.001$ and not significant (ns) (one-way ANOVA).

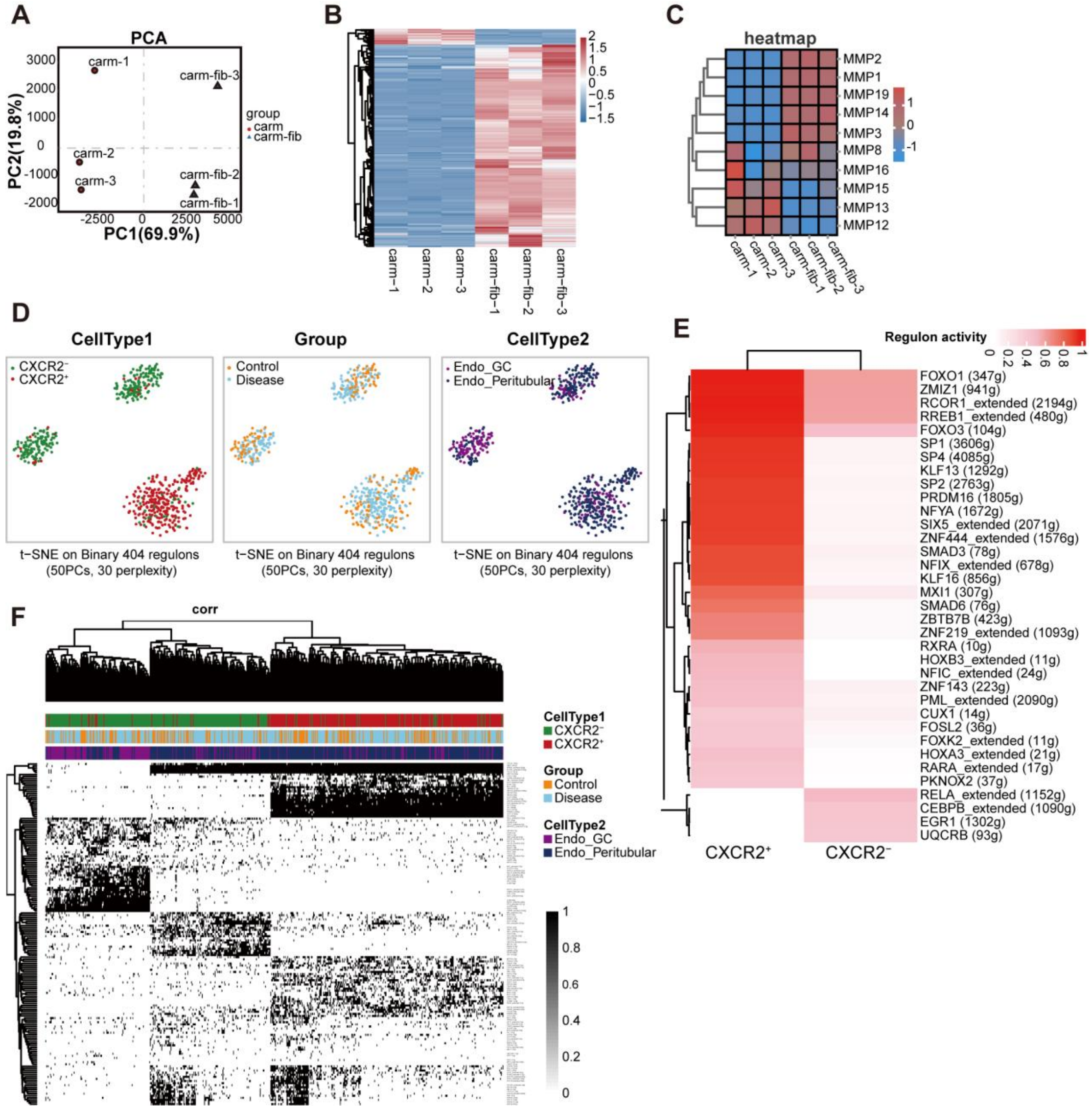

Figure S7. CAR-M2 activates the transcription factor Rxra in Cxcr2^{+} ECs, Related to Figure S7.

(A) PCA dimensionality reduction of transcriptomic differences before and after co-culture of CAR-M2 with FAP^{+} fibroblasts.

(B) Heat map of differentially expressed genes in the transcriptome after CAR-M2 phagocytose FAP^{+} fibroblasts.

(C) Heat map of differential expressions of MMP family genes before and after 24 hours co-culture of CAR-M2 with FAP^{+} fibroblasts.

(D) Re-clustering of endothelial cells from human single-cell sequencing data based on Binary Regulon activity, t-SNE displayed by *CXCR2* expression (left), kidney condition (middle) and EC location (right).

(E) Heat map of differential transcription factor activity between CXCR2^{+} and CXCR2^{-} populations.

(F) The heat map shows changes in transcription factor activity clustered by CXCR2 expression, kidney condition, and EC location.

Supplementary Table 1. Primer sequences for qRT-PCR used in this study.

Gene	Forward sequence 5'-3'	Backward sequence 5'-3'
FAP	ATGAGCTTCCTCGTCCAATTCA	AGACCACCAGAGAGCATATTTTG
CAR-M2	GAAACCTTCTGCAGGGCTGC	AGCGTAAAAGGAGCAACATAG
Il4	TGTAGACCATGTAGTTGAGGTCA	CTGGTTGGCTTCCTTCAC
Cd86	CCATCAGCTTGTCTGTTTCATTCC	GCTGTAATCCAAGGAATGTGGTC
Cd163	CCAGAAGGAACTTGTAGCCACAG	CAGGCACCAAGCGTTTTGAGCT
Arg1	GCCAAGTCCAGAACCATAGG	AAGCAGACCAGCCTTTCTCA
Gapdh	AGGTCGGTGTGAACGGATTTG	TGTAGACCATGTAGTTGAGGTCA
Col1a1	GAGGGCCAAGACGAAGACATC	CAGATCACGTCACGCACAAC
Col3a1	GGAGCTGGCTACTTCTCGC	GGGAACATCCTCCTTCAACAG
Pdgfa	GCAAGACCAGGACGGTCATTT	GGCACTTGACACTGCTCGT
Snail	TCGGAAGCCTAACTACAGCGA	AGATGAGCATTGGCAGCGAG
β-actin	CGTGCGTGACATCAAAGAGAAG	CAAGAAGGAAGGCTGGAAAAGA

Supplementary Table 2. The sequence information of CAR-M2.

Name	Amino acid sequence
anti-FAP scfv	DVLMTQTPLWLPVSLGDQASISCRSSQSIVHSNGNTYLEWYLQKPGQSPKL LIYKVSNEASASGSGGGGSGGGGSQVQLKESGGLVQPGGSLKLSCAASGFT FSSYGMSWVRQTADKRLELVATTNNNGGVTYYPDSVKGRFTISRDNAKNT LYLQMSSLQSEDTAMYYCARYGYYAMDYWGQGISVTVSS
CD8 Hinge+TM	TTTKPVLRTPSPVHPTGTSQPQRPEDCRPRGSVKGTGLDFACDIYIWAPLAGI CVALLLSLIITLIC
4-1BB	SVLKWIRKKFPHIFKQPFKKTTGAAQEEDACSCRCPQEEEGGGGGYEL
CD3ζ	RAKFSRSAETAANLQDPNQLYNELNLGRREEYDVLEKKRARDPEMGGKQQ RRRNPQEGVYNALQKDKMAEAYSEIGTKGERRRGKGHDGLYQGLSTATK DTYDALHMQTLAPR
IL4	MGLNPQLVVILLFFLECTRSHIHGCDKNHLREIIGILNEVTGETPCTEMDVPN VLTATKNTTESELVCRASKVLRIFYLKHGKTPCLKKNSSVLMELQRLFRAF RCLDSSISCTMNESKSTSLKDFLESLKSIMQMDYS
